# Supplementary material for: mRNA vaccination using peptide nanoparticles triggers a strong immune response against endogenous GPC2 in a murine neuroblastoma model
Source: Mol Ther Oncol. 2026 Jun 18;34(2):201244. doi: 10.1016/j.omton.2026.201244 (PMC13293994; doi:10.1016/j.omton.2026.201244)
Supplement: Document S1. Figures S1–S9 and Tables S1 and S2 [file mmc1.pdf]

OMTON, Volume *34*

Supplemental information

mRNA vaccination using peptide nanoparticles triggers a strong immune response against endogenous GPC2 in a murine neuroblastoma model

Ellen King, Chayanika Saha, Rabia Saleem, Binyumeng Jiang, Eve O'Donoghue, Federica Cottone, Helen O. McCarthy, and Olga Piskareva

Table S1. Comparison of RALA-encapsulated NPs

Cargo	N:P ratio	Hydrodynamic size (nm)	Zeta potential (mV)	PdI	Encapsulation Efficiency	Morphology (TEM)	Ref
**mCoV-2**	9	RALA-SME 3.4μg= 29.7 nm<br>RALA-SME 6.7μg = 30.9 nm<br>RALA-SME 26.9μg= 22.1 nm	RALA-SME 3.4μg= 29.7 mV<br>RALA-SME 6.7μg = 30.9 mV<br>RALA-SME 26.9μg = 22.1 mV	RALA-SME 3.4 μg= 0.23<br>RALA-SME 6.7μg = 0.21<br>RALA-SME 26.9μg = 0.23	> 99%	Spherical	[1]
**1) pFKBPL<br>2) siFKBPL**	10	55–65 nm	20–25 mV	< 0.6	/	Spherical	[2]
**miR-26a**	8	Fresh nanoparticles 133.9 nm<br><br>Lyophilised nanoparticles 84.77 nm	25/30 mV	Fresh nanoparticles : 0.33<br><br>Lyophilised nanoparticles : 0.276	Fresh nanoparticles: 81.8%<br><br>Lyophilised nanoparticles: 83.1%	Spherical	[3]
**1)P_4-SedU_2<br>2)P4-SeT2**	RALA/ P_4-SedU_2 2<br>RALA/ P4-SeT2 5	RALA/ P_4-SedU_2 103.3 nM<br>RALA/ P4-SeT2 138.4 nm	RALA/ P_4-SedU_2 18.1 mV<br>RALA/ P4-SeT2 22.6 mV	RALA/ P_4-SedU_2 0.247<br>RALA/ P4-SeT2 0.391	/	Spherical	[4]
**1)pDNA encoding CRISPR machinery and reporter genes<br>2)dCas9-VPR<br>3)Cas9 protein pre-complexed with gRNAs**	1) 10<br>2) 15<br>3)100x	< 150 nm	20 – 30 mV	≥ 0.2	/	Spherical	[5]

**Alendronate**	10	< 100 nm	16 mV	Fresh nanoparticles<br>:<br>0.52<br><br>Lyophilised nanoparticles<br>:<br>0.34	> 95%	Spherical	6
**E6/E7**	10	150 nm	23 mV	< 0.3	>90%	Spherical	7
**RIS**	1	< 100 nm	Between 10 mV and 30 mV	< 0.2	> 90%	Spherical	8
**iNOS**	10	60 nm	25 mV	/	/	Spherical	9
**α-TCP**	5	43 nm	26 mV	/	/	Spherical	10
**PLA-PEG**	10	70.6±7.5 nm	25 mV	0.240±0.054	> 80%	Spherical	11
**siMMP-9**	9	100–110 nm	37.5 mV to 39.8 mV	< 0.35	/	Spherical	12
**pEGFP-N1**	10	126.1 ± 11.9 nm	18.3 ± 4.1 mV	/	/	Spherical	13
**pEGFP-N1**	10	spray-dried 140 to 307 nm	Spry-dried 18 to 37 mV	/	From 25% to 83%, most > 65%	Spherical	14
**1)pEGFP-N1<br>2)PLUX<br>3) HPV-16 E6/E7**	6	Fresh nanoparticles 37.96 nm<br><br>Lyophilised nanoparticles 74.93 nm	Fresh nanoparticles 17.92 mV<br><br>lyophilised nanoparticles 11.77 mV	/	80% at N:P 1, decreasing to about 20% at N:P ratios ≥2,	Spherical	15

**pPSCA**	10	47.27 to 93.09	4.53 to 13.57 mV at N:P ratios ≥4, stabilising as the N:P ratio increased.	/	90%	Spherical	16
**pEGFP-N1**	10	70.6 ± 7.5 nm	+34.47 ± 3.19 mV	0.24 ± 0.05	/	Spherical	17
**pCMV-Luc**	10	100 nm	10-30 mV	/	/	Spherical	18
**EGFP-N1**	10	51 nm	29 mV	0.35	>90%	Spherical	19
**EGFP-N1**	10	187.13 nm	20 mV	0.44	90%	Spherical	20
**siFKBPL**	6	76.6 nm	16.5 mV	/	/	Spherical	21
**sFlt-1-e15a**	10	88 nm	30 mV	/	/	Spherical	22
**α-TCP**	5	100 nm	< 30mV	/	/	Spherical	23
**RALA/ mRNA eGFP**	10	91 nm	26.3 mV	/	88%	Spherical	24

Table S2. Critical Quality Attributes of Nanoparticles for Drug Delivery.

Characteristic	***Acceptable***	***Exceptional***	***Pfizer/BioNTech*** [25,26]	***Moderna*** [27]
**Hydrodynamic Size (nm)**	< 200	< 100	80-100	~220
**Zeta Potential (Charge) (mV)**	~ 20	~ 20	Neutral	Neutral

**Polydispersity Index (PdI)**	< 0.4	< 0.2	~0.2	~0.2

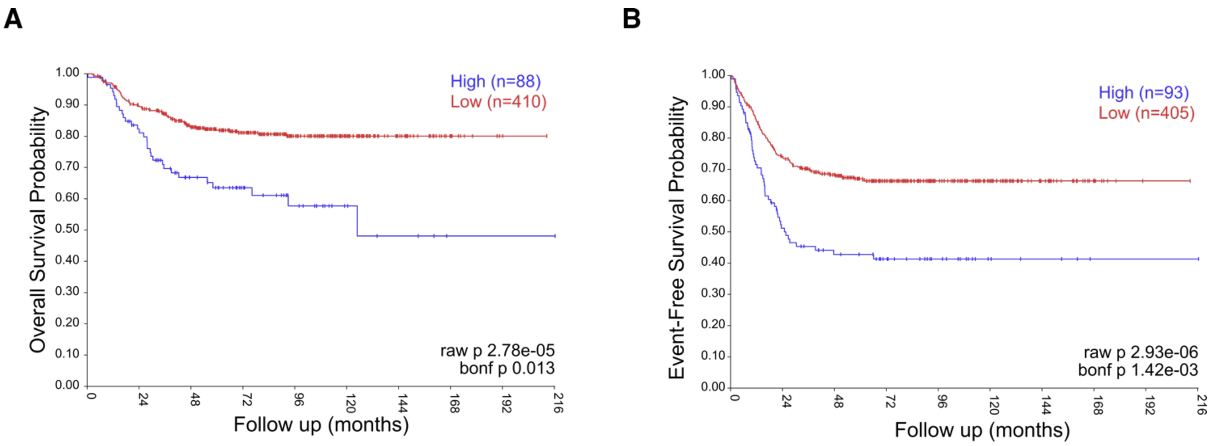

Figure S1. High GPC2 expression correlates with worse overall and event-free survival in a cohort of neuroblastoma patients.
Using Kaplan Meier survival analysis, the significance of high and low GPC2 expression in the SEQC dataset (n=498, [28]) of neuroblastoma patient tumours was assessed based on overall survival (A) and event-free survival (B) in R2: Genomics Analysis and Visualisation Platform (http://r2.amc.nl).

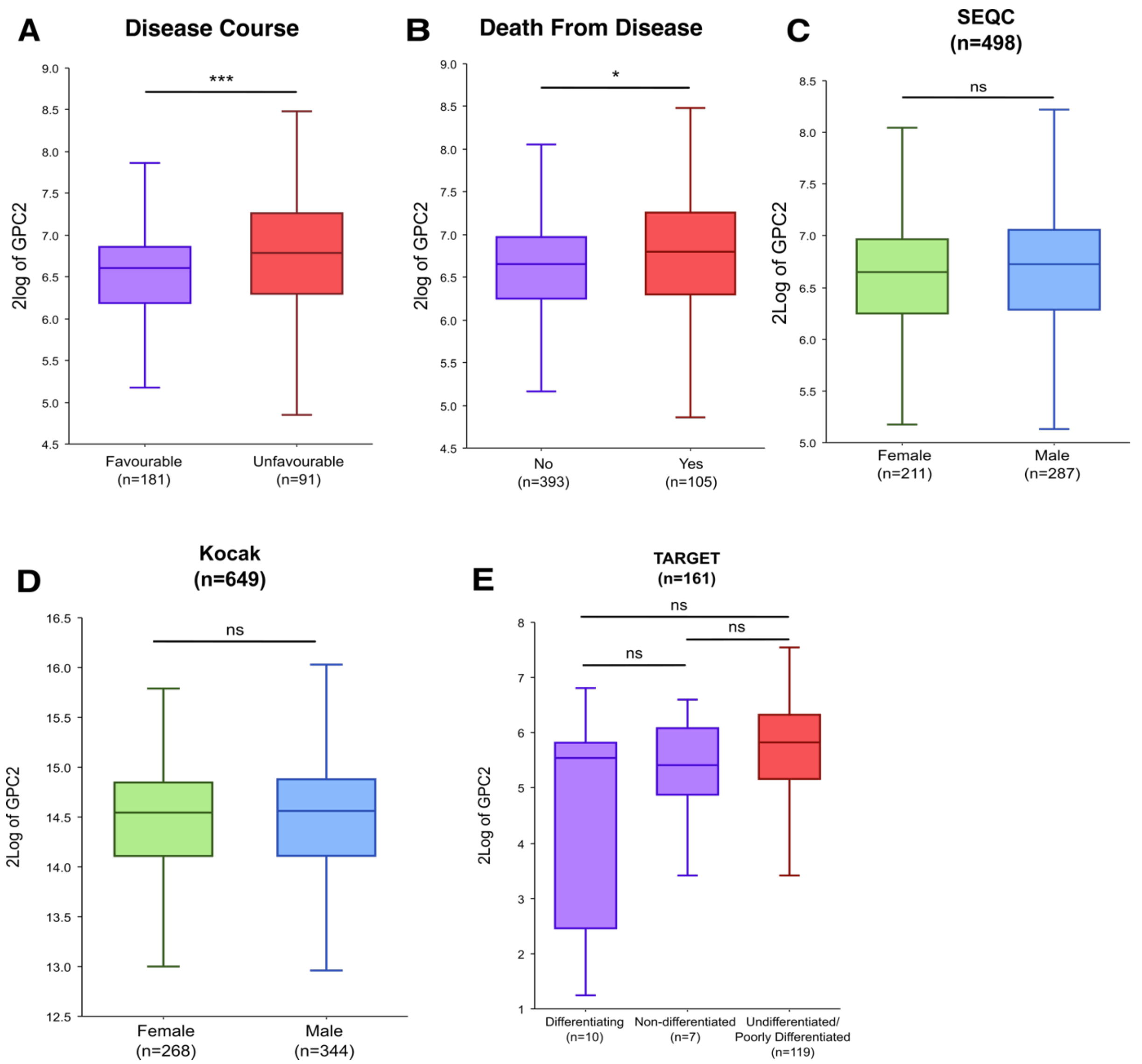

Figure S2. *GPC2* expression analysis vs clinical outcome. (A) disease course and (B) death from disease were examined and correlated with *GPC2* expression. All indicators predictive of adverse clinical outcome, highlighted in red, significantly correlated with increased *GPC2* expression. *GPC2* expression was compared with indicators of clinical outcome using the SEQC dataset (n=498, [28]) in R2: Genomics Analysis and Visualisation Platform (http://r2.amc.nl). Using the (C) SEQC (n=498) and (D) Kocak (n=649) datasets with R2, GPC2 expression was correlated with the gender of the patients, male or female. (E) The association of GPC2 expression with tumour differentiation status was analysed using the TARGET dataset (n=161), with the undifferentiated/poorly differentiated subset, highlighted in red. To determine the significance of GPC2 expression between subsets in each indicator, unpaired t-tests were performed. (*$p \leq 0.05$; **$p \leq 0.01$; ***$p \leq 0.001$; ****$p \leq 0.0001$)

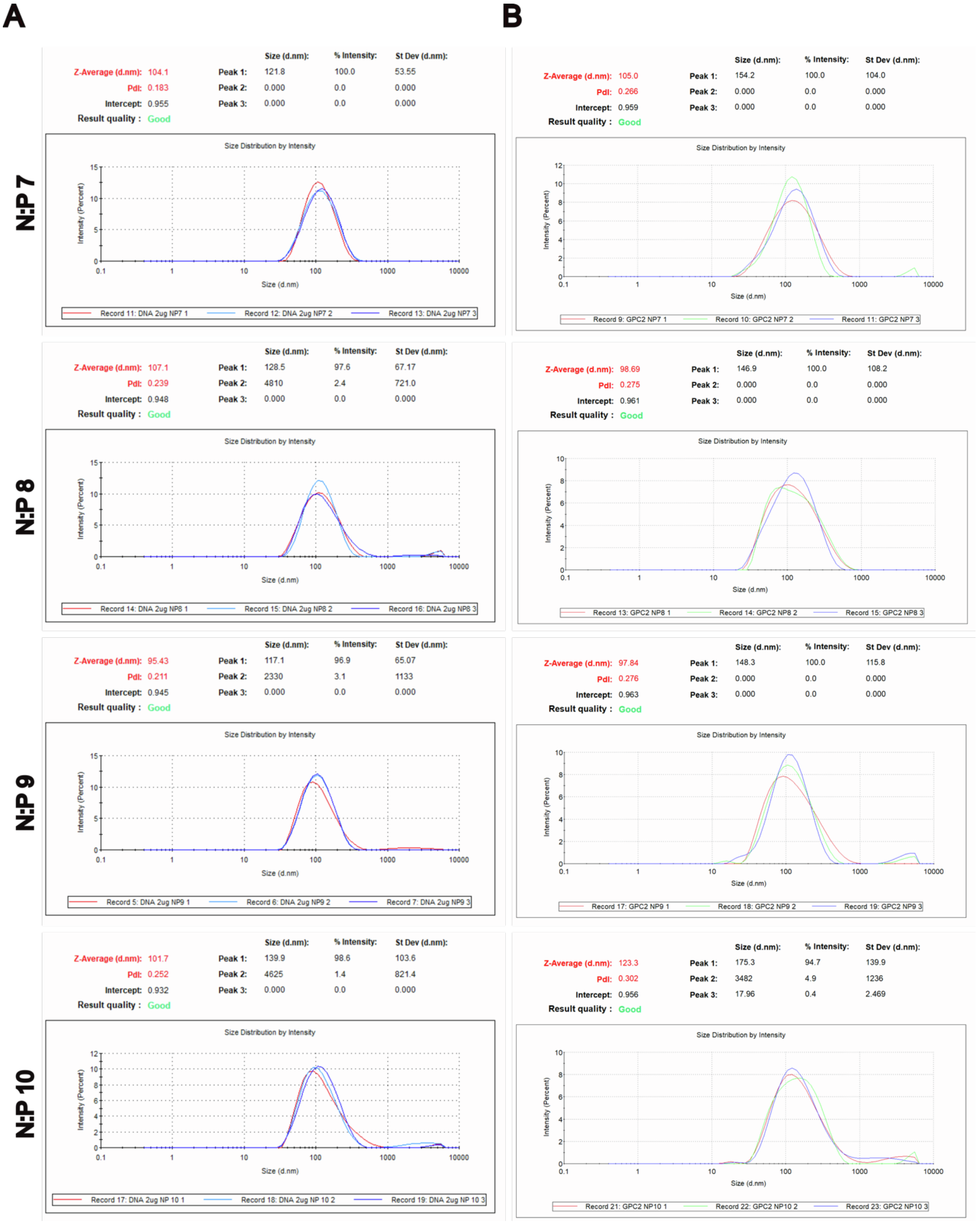

Figure S3. Physicochemical characterisation of RALA/pDNA and RALA/mRNA nanoparticle. Representative size distribution curves of RALA/pGPC2 and RALA/mGPC2 nanoparticles of N:P ratio 7-10. Each curved line represents one repeat out of technical triplicate measurements (N = 3).

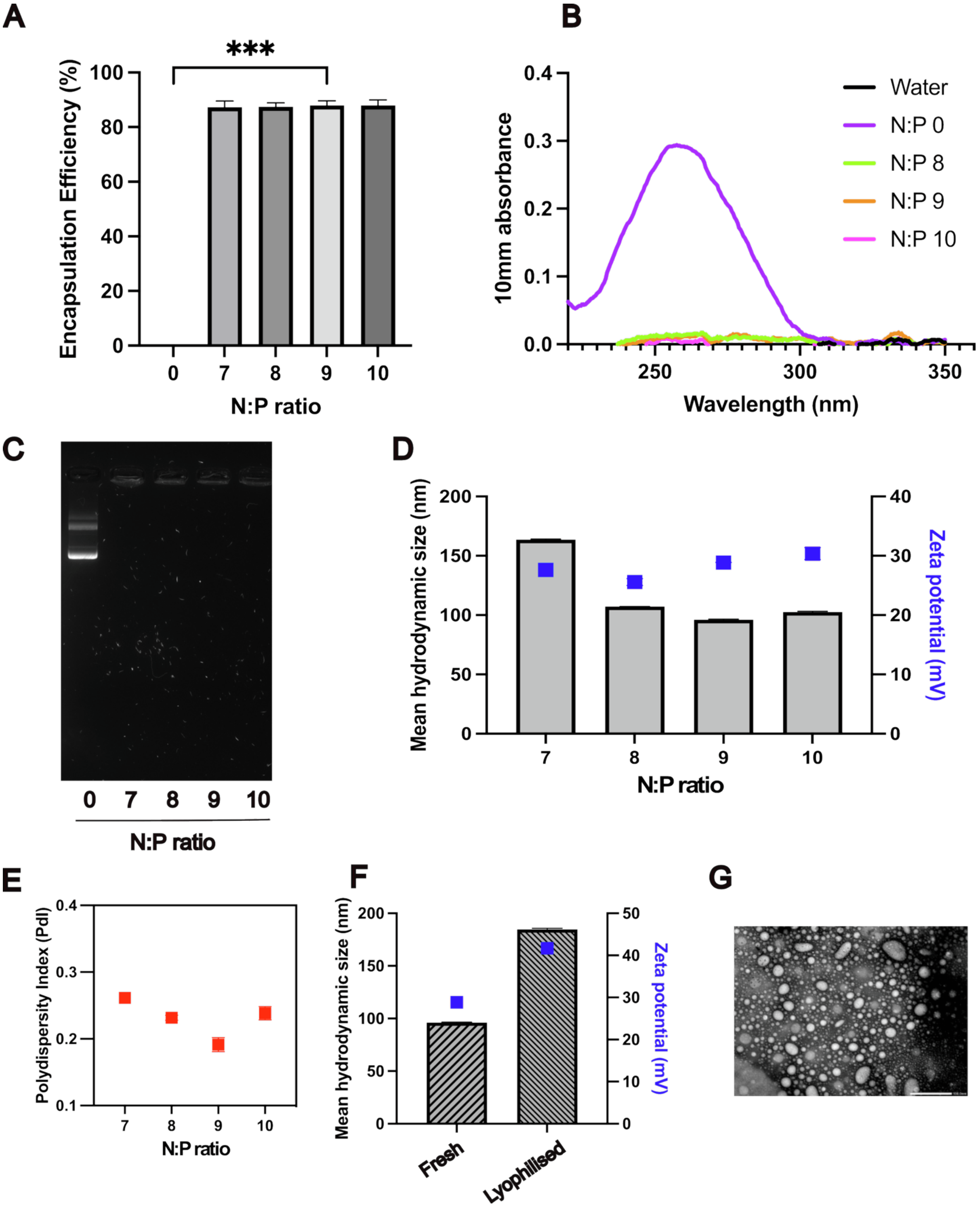

Figure S4. Encapsulation efficiency of RALA/pGPC2 nanoparticles. Encapsulation efficiency of N:P ratios 7-10 was analysed using ion-exchange chromatography (A), PicogreenTM (pGPC2) (B) and agarose gel-shift assay (C). Complexes were incubated at room temperature for 30 min followed by measurement of hydrodynamic size, zeta potential (D) and polydispersity index (E) using a Malvern Zetasizer Nano ZS instrument. Comparison of characteristics of handheld and lyophilised RALA/pGPC2 nanoparticles at N:P ratio 9 (F). Hydrodynamic size, zeta potential and polydispersity index were measured using a Malvern Zetasizer Nano ZS instrument. (N = 3 ± SEM). Statistical analysis was carried out by multiple unpaired T tests, assuming normal distribution. RALA/*pGPC2* NPs were prepared at N:P 9 at a concentration of 1μg/μL and loaded onto carbon-coated copper 400 mesh grids (TAAB Laboratories, UK) (G). Following drying, the samples were stained with 5% uranyl acetate in methanol at room temperature for 1 min, washed with 50% ethanol then molecular grade water

and allowed to dry again. NPs were imaged using a JEM-1400Plus Transmission Electron Microscope (Joel, USA) at an accelerating voltage of 120 kV. Scale bars: 100 nm.

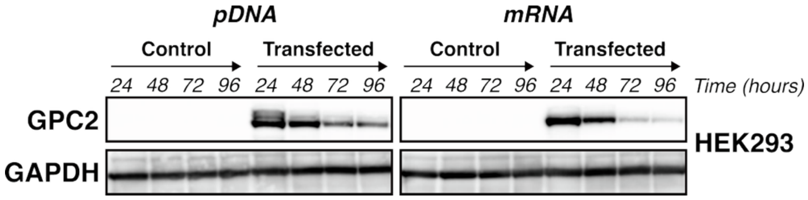

Figure S5. RALA-encapsulated *pGPC2* and *mGPC2* transfection of HEK293 cells at various timepoints.
HEK293 cells were transfected with 1μg of RALA/*pGPC2* and RALA/*mGPC2* NPs for 24, 48, 72 and 96 h. Total protein was isolated and subjected to Western blot analysis to identify GPC2 (~63kDa) overexpression. GAPDH (~36kDa) was used a loading control.

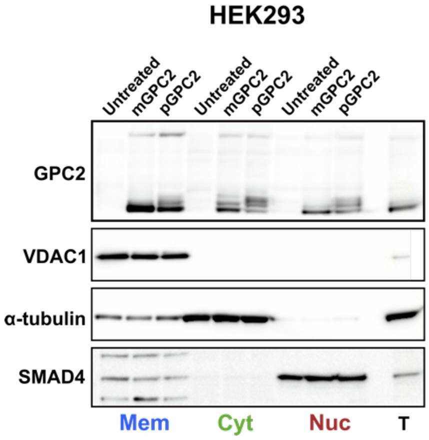

Figure S6. Western blot analysis of HEK293 cells transfected with RALA/pGPC2 and RALA/mGPC2 following subcellular fractionation.
Cells were transfected with 1ug of RALA/mGPC2 and RALA/pGPC2 NPs for 24 and 48 h. Protein from membrane, cytoplasmic and nuclear fractions was isolated and subjected to Western blot analysis to identify GPC2 (~63kDa) overexpression. VDAC1 (30-37kDa), a-tubulin (50kDa) and SMAD4 (65kDa) were used as loading controls for each fraction, respectively.

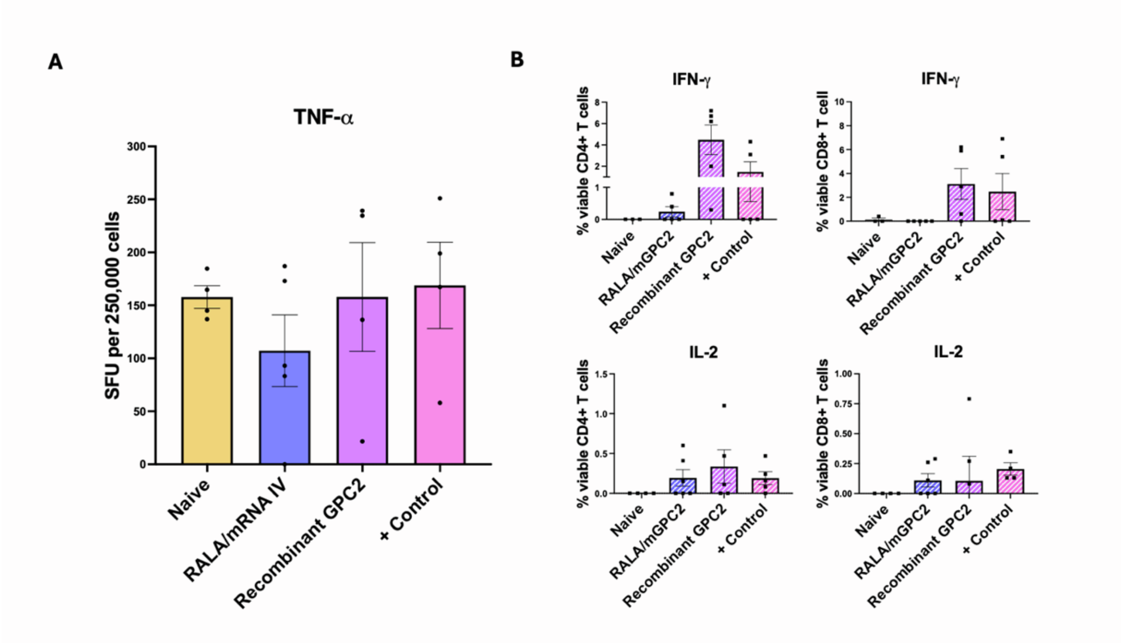

Figure S7. ELISpot analysis of TNF-α, IFN-γ,γ and IL-2 release by splenocytes. ELISpot analysis of cytokine secretion from splenocytes isolated from mice vaccinated with in-house positive control (+ Control), recombinant GPC2, RALA-encapsulated *mGPC2* (RALA/*mGPC2*) compared to unvaccinated mice (Naïve). Vaccinations were delivered i.v. Spot-forming units were counted using Mabtech software. The data shown is an average of technical triplicates and normalized to media-only controls. Negative values are set to zero, and outliers have been removed via ROUT analysis (Q = 5%) using GraphPad PRISM. N ≥ 3 per group. To evaluate statistical differences, an Ordinary one-way ANOVA with Turkey's multiple comparisons was carried out using GraphPad PRISM. Gaussian distribution and standard deviation were assumed based on parametric testing (**p* ≤ 0.05; ***p* ≤ 0.01; ****p* ≤ 0.001; *****p* ≤ 0.0001).

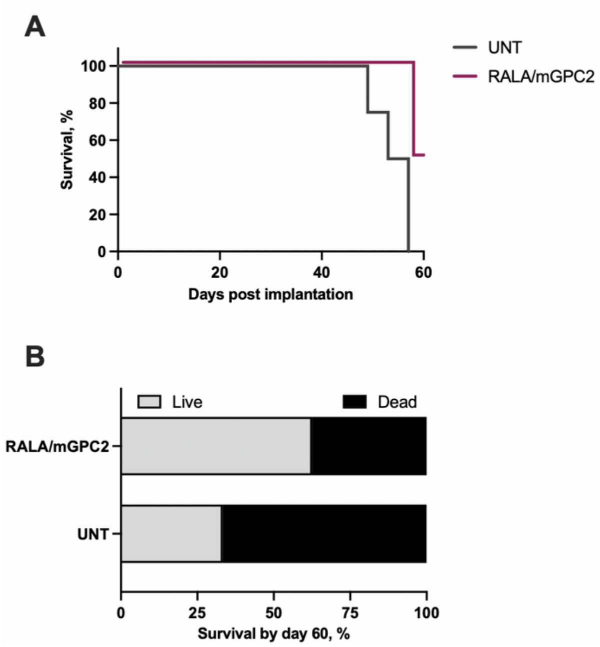

Figure S8. Survival analysis of RALA/mGPC2 vaccinated and unvaccinated mice by day 60. (A) Kaplan-Meier survival analysis; (B) Proportion of live and dead animals.

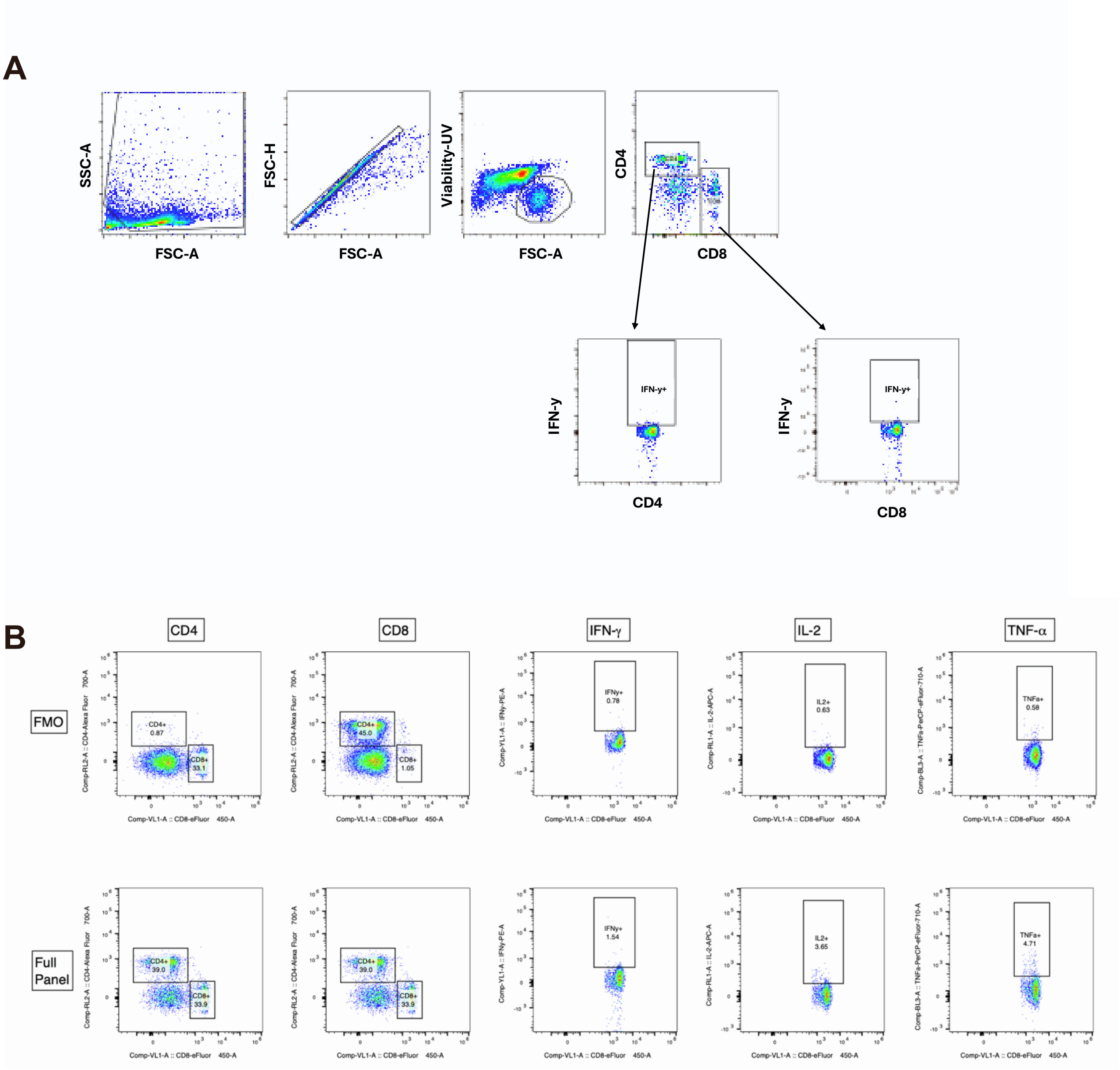

Figure S9. Gating strategy (A) and Fluorescence Minus One (FMO) controls for IFN-γ (B).

References:

1. McCrudden, C.M., Bennie, L., Chambers, P., Wilson, J., Kerr, M., Ziminska, M., Douglas, H., Kuhn, S., Carroll, E., O’Brien, G., et al. (2023). Peptide delivery of a multivalent mRNA SARS-CoV-2 vaccine. Journal of Controlled Release *362*, 536–547. https://doi.org/10.1016/j.jconrel.2023.08.053.
2. Bennett, R., Yakkundi, A., McKeen, H.D., McClements, L., McKeogh, T.J., McCrudden, C.M., Arthur, K., Robson, T., and McCarthy, H.O. (2015). RALA-mediated delivery of FKBPL nucleic acid therapeutics. Nanomedicine *10*, 2989–3001. https://doi.org/10.2217/nnm.15.115.
3. Sadowska, J.M., Ziminska, M., Ferreira, C., Matheson, A., Balouch, A., Bogle, J., Wojda, S., Redmond, J., Elkashif, A., Dunne, N., et al. (2023). Development of miR-26a-activated scaffold to promote healing of critical-sized bone defects through angiogenic and osteogenic mechanisms. Biomaterials *303*, 122398. https://doi.org/10.1016/j.biomaterials.2023.122398.
4. Wilson, J.J., Bennie, L., Eguaogie, O., Elkashif, A., Conlon, P.F., Jena, L., McErlean, E., Buckley, N., Englert, K., Dunne, N.J., et al. (2024). Synthesis and characterisation of a nucleotide based pro-drug formulated with a peptide into a nano-chemotherapy for colorectal cancer. Journal of Controlled Release *369*, 63–74. https://doi.org/10.1016/j.jconrel.2024.03.036.
5. Graham, J.P., Castro, J.G., Werba, L.C., Fardone, L.C., Francis, K.P., Ramamurthi, A., Layden, M., McCarthy, H.O., and Gonzalez-Fernandez, T. (2024). Versatile Cell Penetrating Peptide for Multimodal CRISPR Gene Editing in Primary Stem Cells. Preprint, https://doi.org/10.1101/2024.09.23.614499 https://doi.org/10.1101/2024.09.23.614499.
6. Jena, L.N., Bennie, L.A., McErlean, E.M., Pentlavalli, S., Glass, K., Burrows, J.F., Kett, V.L., Buckley, N.E., Coulter, J.A., Dunne, N.J., et al. (2021). Exploiting the anticancer effects of a nitrogen bisphosphonate nanomedicine for glioblastoma multiforme. J. Nanobiotechnology *19*, 127. https://doi.org/10.1186/s12951-021-00856-x.
7. Ali, A.A., McCrudden, C.M., McCaffrey, J., McBride, J.W., Cole, G., Dunne, N.J., Robson, T., Kissenpfennig, A., Donnelly, R.F., and McCarthy, H.O. (2017). DNA vaccination for cervical cancer; a novel technology platform of RALA mediated gene delivery via polymeric microneedles. Nanomedicine *13*, 921–932. https://doi.org/10.1016/j.nano.2016.11.019.
8. Glass, K., Fines, C., Coulter, P., Jena, L., McCarthy, H.O., and Buckley, N. (2024). Development and Characterization of a Peptide-Bisphosphonate Nanoparticle for the Treatment of Breast Cancer. Mol. Pharm. *21*, 4970–4982. https://doi.org/10.1021/acs.molpharmaceut.4c00299.
9. McCrudden, C.M., McBride, J.W., McCaffrey, J., Ali, A.A., Dunne, N.J., Kett, V.L., Coulter, J.A., Robson, T., and McCarthy, H.O. (2017). Systemic RALA/iNOS Nanoparticles: A Potent Gene Therapy for Metastatic Breast Cancer Coupled as a Biomarker of Treatment. Mol. Ther. Nucleic Acids *6*, 249–258. https://doi.org/10.1016/j.omtn.2016.12.010.
10. O’Doherty, M., Mulholland, E.J., Chambers, P., Pentlavalli, S., Ziminska, M., Chalanqui, M.J., Pauly, H.M., Sathy, B.N., Donahue, T.H., Kelly, D.J., et al. (2020). Improving the Intercellular Uptake and Osteogenic Potency of Calcium Phosphate via Nanocomplexation with the RALA Peptide. Nanomaterials *10*, 2442. https://doi.org/10.3390/nano10122442.
11. Jain, A.K., Massey, A., Yusuf, H., Kett, V.L., McDonald, D., and McCarthy, H. (2015). Development of polymeric–cationic peptide composite nanoparticles, a

nanoparticle-in-nanoparticle system for controlled gene delivery. Int. J. Nanomedicine, 7183. https://doi.org/10.2147/IJN.S95245.
12. Yan, L.-P., Castaño, I.M., Sridharan, R., Kelly, D., Lemoine, M., Cavanagh, B.L., Dunne, N.J., McCarthy, H.O., and O'Brien, F.J. (2020). Collagen/GAG scaffolds activated by RALA-siMMP-9 complexes with potential for improved diabetic foot ulcer healing. Materials Science and Engineering: C *114*, 111022. https://doi.org/10.1016/j.msec.2020.111022.
13. Ziminska, M., Wilson, J.J., McErlean, E., Dunne, N., and McCarthy, H.O. (2020). Synthesis and Evaluation of a Thermoresponsive Degradable Chitosan-Grafted PNIPAAm Hydrogel as a "Smart" Gene Delivery System. Materials *13*, 2530. https://doi.org/10.3390/ma13112530.
14. Munir, M., Kett, V.L., Dunne, N.J., and McCarthy, H.O. (2022). Development of a Spray-Dried Formulation of Peptide-DNA Nanoparticles into a Dry Powder for Pulmonary Delivery Using Factorial Design. Pharm. Res. *39*, 1215–1232. https://doi.org/10.1007/s11095-022-03256-4.
15. Cole, G., Ali, A.A., McCrudden, C.M., McBride, J.W., McCaffrey, J., Robson, T., Kett, V.L., Dunne, N.J., Donnelly, R.F., and McCarthy, H.O. (2018). DNA vaccination for cervical cancer: Strategic optimisation of RALA mediated gene delivery from a biodegradable microneedle system. European Journal of Pharmaceutics and Biopharmaceutics *127*, 288–297. https://doi.org/10.1016/j.ejpb.2018.02.029.
16. Cole, G., Ali, A.A., McErlean, E., Mulholland, E.J., Short, A., McCrudden, C.M., McCaffrey, J., Robson, T., Kett, V.L., Coulter, J.A., et al. (2019). DNA vaccination via RALA nanoparticles in a microneedle delivery system induces a potent immune response against the endogenous prostate cancer stem cell antigen. Acta Biomater. *96*, 480–490. https://doi.org/10.1016/j.actbio.2019.07.003.
17. Jain, A.K., Yusuf, H., Pattani, A., McCarthy, H.O., McDonald, D.M., and Kett, V.L. (2014). Development of a method to quantify the DNA content in cationic peptide–DNA nanoparticles. J. Pharm. Biomed. Anal. *100*, 236–242. https://doi.org/10.1016/j.jpba.2014.07.036.
18. McCaffrey, J., McCrudden, C.M., Ali, A.A., Massey, A.S., McBride, J.W., McCrudden, M.T.C., Vicente-Perez, E.M., Coulter, J.A., Robson, T., Donnelly, R.F., et al. (2016). Transcending epithelial and intracellular biological barriers; a prototype DNA delivery device. Journal of Controlled Release *226*, 238–247. https://doi.org/10.1016/j.jconrel.2016.02.023.
19. McCarthy, H.O., McCaffrey, J., McCrudden, C.M., Zholobenko, A., Ali, A.A., McBride, J.W., Massey, A.S., Pentlavalli, S., Chen, K.-H., Cole, G., et al. (2014). Development and characterization of self-assembling nanoparticles using a bio-inspired amphipathic peptide for gene delivery. Journal of Controlled Release *189*, 141–149. https://doi.org/10.1016/j.jconrel.2014.06.048.
20. McErlean, E.M., McCrudden, C.M., McBride, J.W., Cole, G., Kett, V.L., Robson, T., Dunne, N.J., and McCarthy, H.O. (2021). Rational design and characterisation of an amphipathic cell penetrating peptide for non-viral gene delivery. Int. J. Pharm. *596*, 120223. https://doi.org/10.1016/j.ijpharm.2021.120223.
21. Mulholland, E.J., Ali, A., Robson, T., Dunne, N.J., and McCarthy, H.O. (2019). Delivery of RALA/siFKBPL nanoparticles via electrospun bilayer nanofibres: An innovative angiogenic therapy for wound repair. Journal of Controlled Release *316*, 53–65. https://doi.org/10.1016/j.jconrel.2019.10.050.
22. McNally, R., Alqudah, A., McErlean, E.M., Rennie, C., Morshed, N., Short, A., McGrath, K., Shimoni, O., Robson, T., McCarthy, H.O., et al. (2021). Non-Viral Gene Delivery

Utilizing RALA Modulates sFlt-1 Secretion, Important for Preeclampsia. Nanomedicine *16*, 1999–2012. https://doi.org/10.2217/nnm-2021-0180.
23. Sathy, B.N., Olvera, D., Gonzalez-Fernandez, T., Cunniffe, G.M., Pentlavalli, S., Chambers, P., Jeon, O., Alsberg, E., McCarthy, H.O., Dunne, N., et al. (2017). RALA complexed α-TCP nanoparticle delivery to mesenchymal stem cells induces bone formation in tissue engineered constructs in vitro and in vivo. J. Mater. Chem. B *5*, 1753–1764. https://doi.org/10.1039/C6TB02881K.
24. Udhayakumar, V.K., De Beuckelaer, A., McCaffrey, J., McCrudden, C.M., Kirschman, J.L., Vanover, D., Van Hoecke, L., Roose, K., Deswarte, K., De Geest, B.G., et al. (2017). Arginine-Rich Peptide-Based mRNA Nanocomplexes Efficiently Instigate Cytotoxic T Cell Immunity Dependent on the Amphipathic Organization of the Peptide. Adv. Healthc. Mater. *6*. https://doi.org/10.1002/adhm.201601412.
25. Pardi, N., Tuyishime, S., Muramatsu, H., Kariko, K., Mui, B.L., Tam, Y.K., Madden, T.D., Hope, M.J., and Weissman, D. (2015). Expression kinetics of nucleoside-modified mRNA delivered in lipid nanoparticles to mice by various routes. Journal of Controlled Release *217*, 345–351. https://doi.org/10.1016/j.jconrel.2015.08.007.
26. Kudsiova, L., Lansley, A., Scutt, G., Allen, M., Bowler, L., Williams, S., Lippett, S., Stafford, S., Tarzi, M., Cross, M., et al. (2021). Stability testing of the Pfizer-BioNTech BNT162b2 COVID-19 vaccine: A translational study in UK vaccination centres. BMJ Open Science *5*. https://doi.org/10.1136/bmjos-2021-100203.
27. Hermosilla, J., Alonso-García, A., Salmerón-García, A., Cabeza-Barrera, J., Medina-Castillo, A.L., Pérez-Robles, R., and Navas, N. (2023). Analysing the In-Use Stability of mRNA-LNP COVID-19 Vaccines Comirnaty^{TM} (Pfizer) and Spikevax^{TM} (Moderna): A Comparative Study of the Particulate. Vaccines (Basel). *11*. https://doi.org/10.3390/vaccines11111635.
28. Su, Z., Fang, H., Hong, H., Shi, L., Zhang, W., Zhang, W., Zhang, Y., Dong, Z., Lancashire, L.J., Bessarabova, M., et al. (2014). An investigation of biomarkers derived from legacy microarray data for their utility in the RNA-seq era. Genome Biol. *15*, 523. https://doi.org/10.1186/s13059-014-0523-y.
