# Supplementary material for: mRNA vaccination using peptide nanoparticles triggers a strong immune response against endogenous GPC2 in a murine neuroblastoma model
Source: Mol Ther Oncol. 2026 Jun 18;34(2):201244. doi: 10.1016/j.omton.2026.201244 (PMC13293994; doi:10.1016/j.omton.2026.201244)
Supplement: Document S2. Article plus supplemental information [file mmc2.pdf]

Molecular Therapy
Oncology
Original Article

mRNA vaccination using peptide nanoparticles triggers a strong immune response against endogenous GPC2 in a murine neuroblastoma model

Ellen King,[1,2,3] Chayanika Saha,[4] Rabia Saleem,[1,2,3] Binyumeng Jiang,[4] Eve O'Donoghue,[1,2,3] Federica Cottone,[1,2,3] Helen O. McCarthy,[4,6] and Olga Piskareva[1,2,3,5,6]

[1]Cancer Bioengineering Group, Department of Anatomy and Regenerative Medicine, RCSI University of Medicine and Health Sciences, Dublin, Ireland; [2]School of Pharmacy and Biomolecular Sciences, RCSI, Dublin, Ireland; [3]Tissue Engineering Research Group, Department of Anatomy and Regenerative Medicine, RCSI University of Medicine and Health Sciences, Dublin, Ireland; [4]School of Pharmacy, Queen's University of Belfast, 97 Lisburn Road, Belfast BT9 7BL, UK; [5]Advanced Materials and Bioengineering Research Center (AMBER), RCSI and Trinity College Dublin, Dublin, Ireland

Neuroblastoma is an aggressive pediatric solid tumor that arises during embryonic development and contributes to 15% of cancer-related deaths in children. To date, neither experimental nor clinical trial data on an mRNA vaccine for neuroblastoma have been published, highlighting a significant gap in the anticancer vaccine development pipeline. This study presents the first mRNA vaccine for the treatment of neuroblastoma. We explored the self-assembling peptide RALA for delivering mRNA encoding glypican 2 (GPC2), a potent tumor-associated antigen in neuroblastoma. These data outline rigorous *in vitro* characterization of vaccine nanoparticle formulations, cellular uptake, and functionality. Immunization of mice with RALA/*mGPC2* generated an antigen-specific cellular immune response against GPC2, with significant increases in IFN-γ and IL-2 expression by splenocytes and TNF-α expression by CD4$^+$ and CD8$^+$ T cells. Immunization delayed tumor development by 10–11 days and reduced tumor volume by 70% compared with unvaccinated controls in a subcutaneous murine model of *MYCN*-amplified neuroblastoma. This work demonstrates the therapeutic potential of the RALA/*mGPC2* vaccine for treating neuroblastoma. Additionally, GPC2 is upregulated across multiple adult and pediatric cancer subtypes, establishing this vaccine as an attractive immunotherapy with far-reaching potential.

INTRODUCTION

Neuroblastoma is an aggressive pediatric solid tumor that arises during embryonic development and contributes to 15% of cancer-related deaths in children. Four out of five patients with clinically aggressive disease do not show sustained responses to recent advances in anticancer therapy, thus highlighting the urgent need for novel treatments.[1] Neuroblastoma survivors are at risk of severe or life-threatening illnesses due to late treatment toxicities, including the development of secondary cancers, further emphasizing the need for more targeted therapy.

To develop a clinically relevant neuroblastoma mRNA vaccine, selecting a potent tumor-associated antigen (TAA) is critical. Several immunotherapeutic targets, such as glypican-2 (GPC2), New York esophageal squamous cell carcinoma 1 (NY-ESO-1), B7-H3 (known as CD276), neural cell adhesion molecule (NCAM, known as CD56), L1 cell adhesion molecule (L1CAM, known as CD171), and preferentially expressed in melanoma antigen (PRAME), have demonstrated promise in the neuroblastoma field.[2] The majority of immunotherapies for neuroblastoma have so far focused on a disialoganglioside (GD2) due to its high expression on tumor cells.[3] However, significant neural toxicities have been associated with this therapy due to the expression of GD2 on nerve cells.[4,5]

GPC2, an oncofetal antigen expressed in early development and largely silenced in normal adult tissues, is upregulated in multiple cancer subtypes, including neuroblastoma, glioblastoma, and epithelial solid tumors (Figure 1A, reviewed in Chen et al.[6]). High expression of GPC2 correlates with poor event-free survival, overall survival, and adverse clinical outcomes in neuroblastoma (Figures 1 and S1).[7] Interestingly, GPC2-directed CAR-T cell therapy has advanced to a clinical trial (NCT05650749), with preliminary pre-clinical data demonstrating potent anti-tumor responses and minimal off-tumor toxicities. This suggests that GPC2 is a strong candidate for a neuroblastoma mRNA vaccine.[7,8]

mRNA vaccines hold great promise as they boost patients' immune systems to attack and eliminate tumors. mRNA vaccines have several advantages compared to traditional vaccine platforms, such as those based on viruses or DNA.[9,10] They are relatively easy to produce and can be adapted to treat various diseases. The production is free from

Received 17 December 2025; accepted 21 May 2026;
https://doi.org/10.1016/j.omton.2026.201244.

[6]Senior author

Correspondence: Olga Piskareva, Department of Anatomy and Regenerative Medicine, RCSI University of Medicine and Health Sciences Dublin, Ireland.
E-mail: olgapiskareva@rcsi.ie

© 2026 The Author(s).
Published by Elsevier Inc. on behalf of The American Society of Gene and Cell Therapy.
This is an open access article under the CC BY license (http://creativecommons.org/licenses/by/4.0/).

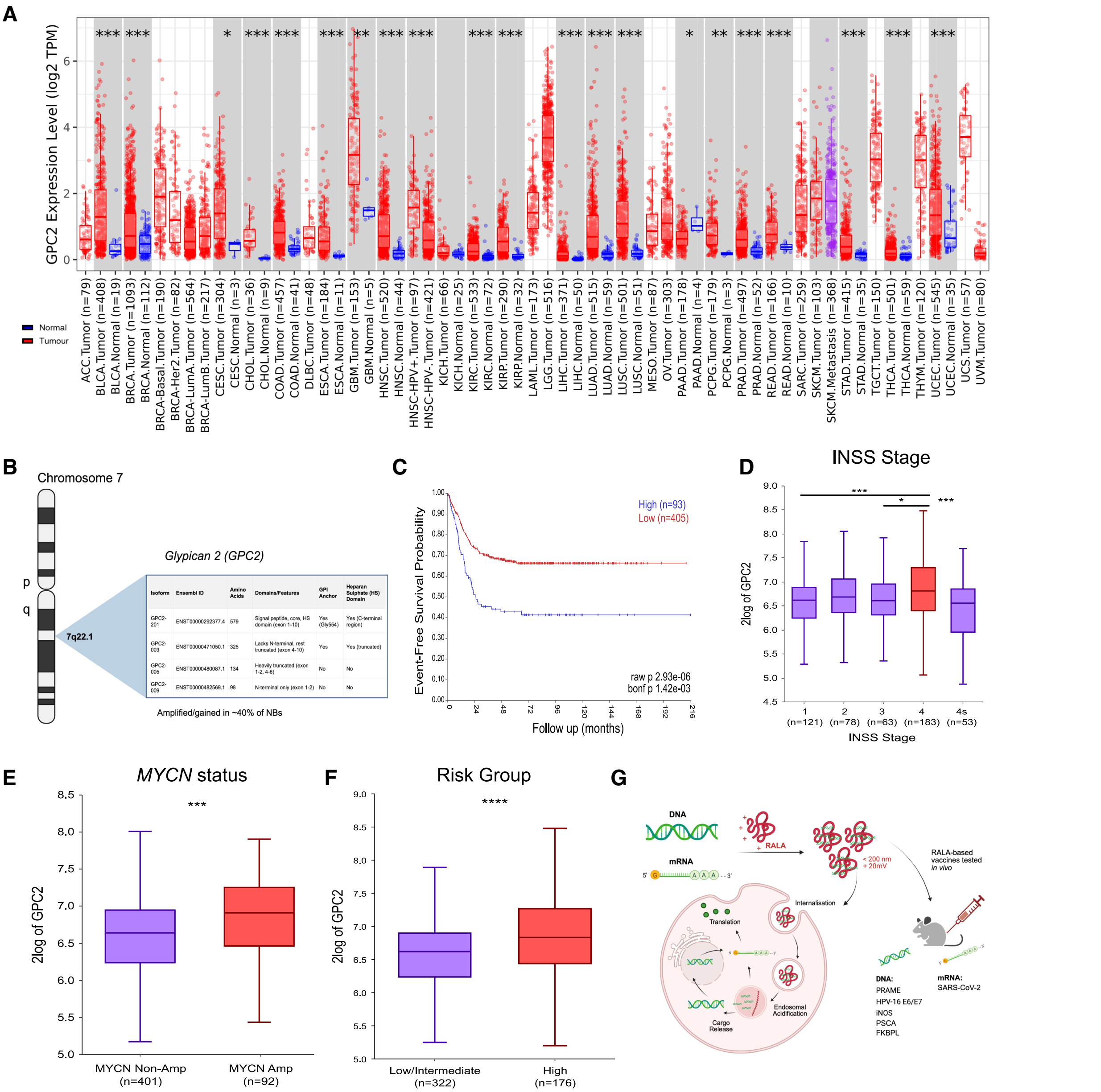

Figure 1. A summary of glypican-2 as a tumor-associated antigen

(A) Differential expression of *GPC2* between tumor and adjacent normal tissues across all the Tissue Cancer Genome Atlas (TCGA) cancer types. The corresponding RNA-seq datasets were uniformly processed via the UALCAN database (http://ualcan.path.uab.edu/). Gene expression levels are displayed as boxplots, and statistical significance was assessed using the Wilcoxon test. (B) Chromosomal localization of *GPC2* and corresponding *GPC2* transcripts. *GPC2* expression was compared with clinical outcome indicators using the SEQC dataset (n = 498) and R2: Genomics Analysis and Visualization Platform (http://r2.amc.nl). (C) Event-free survival, (D) INSS stage, (E) *MYCN*-amplification status, (F) neuroblastoma risk groups—all indicators predictive of adverse clinical outcome, highlighted in red. Boxes with horizontal lines indicate the median with 25th and 75th percentiles, and the largest and smallest values are shown with vertical bars. To determine the significance of *GPC2* expression between subsets in each indicator, unpaired t tests were performed. (*$p \leq 0.05$; **$p \leq 0.01$; ***$p \leq 0.001$; ****$p \leq 0.0001$) (G) RALA-mediated encapsulation and cellular delivery of mRNA and pDNA. Illustrations were created in BioRender.com.

toxic chemicals and virus contamination. mRNA has a negligible risk of genome integration because its natural functions occur in the cytoplasm, unlike DNA- or virus-based vaccines.

RALA is composed of a repeating amino acid sequence of arginine (R), alanine (A), leucine (L), and alanine (A) that self-assembles into stable nanoparticles (NPs), protecting DNA or RNA and

maintaining a stable random coil structure at a physiological pH of 7.4 (Figure 1G). Upon cellular internalization, RALA becomes protonated, increasing the alpha-helicity and facilitating endosomal escape.[11] The safe and efficient delivery of DNA/RNA using RALA technology is well documented, with published therapeutic vaccines targeting antigens, such as spike, membrane, and envelope proteins for COVID-19,[12] prostate stem cell antigen,[12] iNOS,[13] PRAME,[14] and the FK506-binding protein like-FKBPL gene[15] (Figure 1G; Table S1). Compared to other delivery systems, RALA technology offers several advantages: high mRNA encapsulation efficiency, straightforward purification, no immune response to RALA itself, no restriction on the size or number of mRNA cargos to be delivered, stability at room temperature, and lower costs.

Promising data have recently emerged from a phase 2b clinical trial evaluating the cancer vaccine mRNA-4157 (V940) in combination with MSD's Keytruda, an immune checkpoint inhibitor, in patients with resected high-risk melanoma. The basis of mRNA cancer vaccines lies in the generation of an antigen-specific cellular immune response that encompasses both $CD4^+$ and $CD8^+$ T cells. To do so, mRNA is delivered to antigen-presenting cells in secondary lymphoid tissues, where it is translated into protein, processed, and presented as epitopes on MHC class I molecules. Upon release and secondary uptake, a similar presentation occurs on MHC class II molecules, and thus both $CD4^+$ and $CD8^+$ T cells become activated once bound, exerting their effector functions. $CD8^+$ cytotoxic T cells release cytokines, perforin, and granzymes to eliminate tumor cells, and ideally, post-activation, remain circulating as memory cells capable of re-emergence upon re-challenge in the case of tumor recurrence.

Our study presents the first mRNA vaccine for neuroblastoma. This vaccine is composed of mRNA encoding the full-length human *GPC2*, complexed with the RALA peptide, optimally formulated for antigen-presenting cell delivery, and proven to generate an antigen-specific cellular immune response. Key to this work is the significant tumor burden control demonstrated following vaccination of mice. We present the first preclinical study demonstrating anti-tumor responses with a GPC2-directed vaccine in neuroblastoma.

RESULTS

Physiochemical characterization and stability of RALA/*mGPC2* NPs

It has been previously shown that RALA can encapsulate nucleic acids at a variety of N:P ratios (2–12)[12] and that, for most particles, an N:P ratio of 6–12 effectively condenses and encapsulates cargo.[11–13,16–20] As such, RALA/*mGPC2* NPs were synthesized at ratios ranging from 0 to 10 for physicochemical assessment, using RALA/*pGPC2* as a reference (Figure S3). Encapsulation efficiency was assessed by combining nucleic acid binding (Figure 2A), agarose gel electrophoresis (Figure 2B), and ion-exchange chromatography (Figure 2E). This confirmed an encapsulation efficiency of greater than 94%. Dynamic light scattering (DLS) results had an average size of 99.07 nm. RALA/*mGPC2* NPs (N:P 9) had an average positive charge of 26.433 mV and a polydispersity index (PDI) of 0.288, indicating a homogenous population (Figures 2C and 2D). Moving forward with an N:P ratio of 9, we conducted transmission electron microscopy (TEM) to visually assess the shape and size of RALA/*mGPC2* NPs (Figure 2H). The NP suspension showed clear, rounded complexes within the size range previously evaluated using DLS. Size differences between DLS and TEM images are primarily due to the measurement principles of each technique. DLS measures the hydrodynamic environment, including the solvent layer surrounding particles, which can lead to larger reported sizes. In contrast, TEM gives a dry-state measurement of the NP physical core without a hydration shell, typically appearing smaller.[21] Compared to a previously published mRNA vaccine using RALA technology, RALA/*mGPC2* has similar formulation characteristics.[16]

We also compared NP characteristics before and after lyophilization. Lyophilization increased the charge of RALA/*mGPC2* NPs (FC: 6.53 ± 0.5 [p = 0.0002]), however, it also reduced size (FC: 3.6 ± 0.7 [p = 0.0073]) and improved PDI (FC: 0.09 ± 0.02 [p = 0.0048]) (Figure 2F). However, these changes did not alter NP physicochemical attributes outside of critical quality attributes (Table S2).

In vitro assessment of RALA/*mGPC2* NPs

RALA/*mGPC2* NP functionality was evaluated *in vitro* in a dendritic cell line (DC2.4), a popular choice of antigen-presenting cell *in vitro* for vaccines,[22] and a human embryonic kidney cell line (HEK293). Cells were transfected, and total protein was collected at 24, 48, and 72 h and subjected to western blot analysis. GPC2 expression was observed after 24 h of transfection and then quickly subsided, returning to baseline at 72 h (Figures 2G and S5).

RALA/*mGPC2* transfection of DC2.4 cells was repeated, followed by subcellular fractionation to identify the location of GPC2 expression. VDAC1, alpha-tubulin, and SMAD4 were used as loading controls for each fraction. Clear band at ∼60 kDa is visible only in the membrane fraction of RALA/*mGPC2*-transfected DC2.4 cells and HEK293, with no GPC2 expression observed in the nuclear or cytoplasmic fractions (Figures 2I and S6). Similarly, no GPC2 expression was observed in empty vector-transfected controls.

Importantly, cell viability following transfection with RALA complexes was considerably higher (∼90% for RALA/*mGPC2*) at 48 h compared to commercially available agents such as lipofectamine 3000 at ∼20%, demonstrating the low cytotoxicity of RALA (Figure 2J).

Immunization of mice with RALA/*mGPC2* generates a potent cellular immune response against the encoded GPC2

We evaluated the immunogenicity of 20 μg of RALA/*mGPC2* *in vivo* using C57BL/6 mice immunized in a prime-boost regimen via intravenous injection. To understand the antigen-specific cellular immune response driven by vaccination, splenocytes were stimulated *ex vivo* with either media alone or a GPC2 overlapping peptide pool for 48 h. Splenocyte production of IFN-γ and IL-2 was

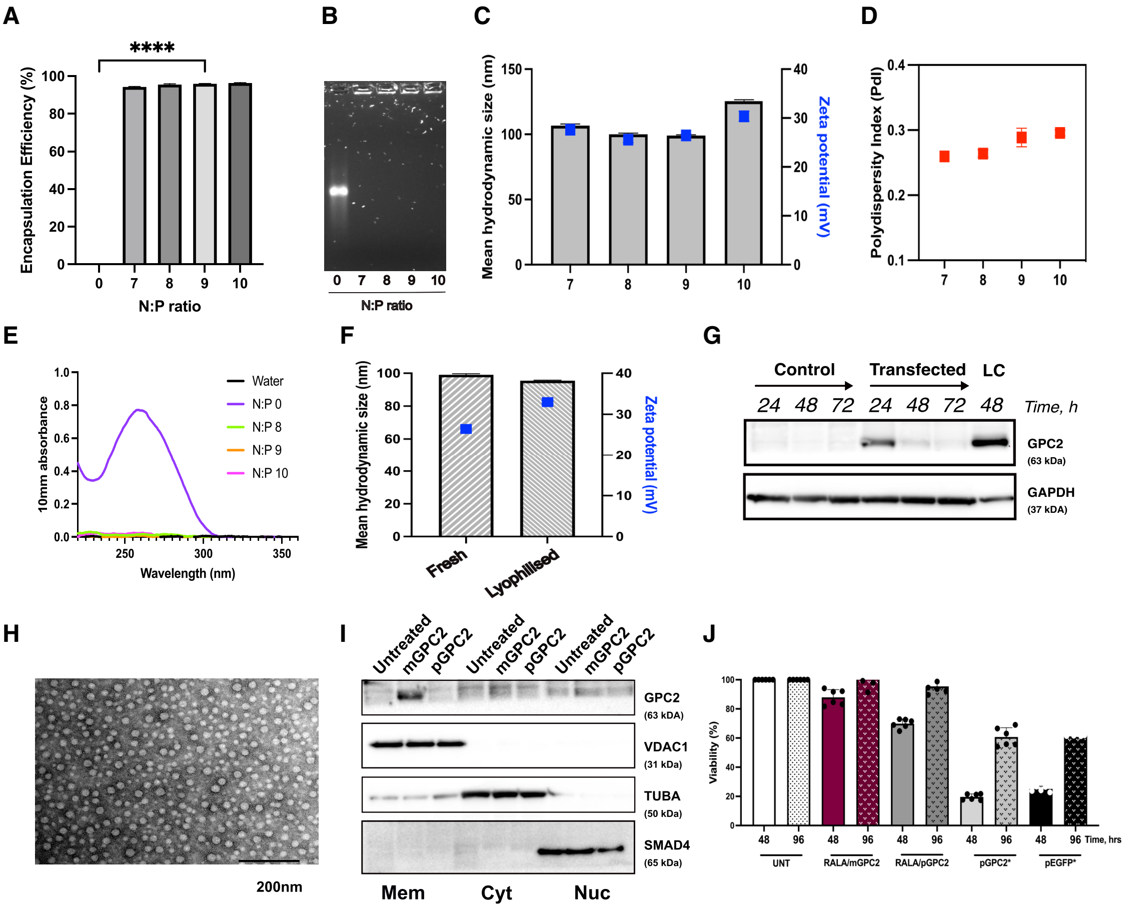

Figure 2. Physicochemical and functional analysis of RALA/*mGPC2* nanoparticles

(A) Encapsulation efficiency of N:P ratios 7–10 was analyzed using RiboGreen (*mGPC2*) assay, agarose gel shift assay (B), and ion-exchange chromatography (E). Hydrodynamic size, zeta potential (C), and polydispersity index (D). (F) Comparison of hydrodynamic size and zeta potential of the fresh-made and lyophilized RALA/*mGPC2* nanoparticles (N:P ratio 9). (G) Western blot analysis of DC2.4 cells transfected with RALA/*mGPC2* NPs for 24, 48, and 72 h (representative image). Control denotes empty vector-transfected DC2.4 cells. LC denotes a positive protein loading control for GPC2. (H) Transmission electron microscopy of RALA/*mGPC2* nanoparticles. Scale bars, 200 nm. (I) Western blot analysis to identify GPC2 (~63 kDa) overexpression in membrane (Mem), cytoplasmic (Cyto), and nuclear (Nuc) fractions of DC2.4 cells transfected with RALA/*mGPC2* NPs for 48 h. VDAC1 (31 kDa), a-tubulin (50 kDa), and SMAD4 (65 kDa) were used as loading controls for each fraction. (J) Assessment of DC2.4 viability 48 and 96 h after transfection with RALA/*mGPC2*, RALA/*pGPC2*, Lipofectamine 3000/*pGPC2*, and Lipofectamine 3000/*pEGFP* NPs. The data represent the means of N3 ± SEMs and were statistically evaluated by one-way ANOVA (A) (****$p \leq 0.0001$). Only statistically significant comparisons are graphed.

measured using the ELISpot assay, and antigen-specific responses were calculated following deduction of spot forming units (SFU) from unstimulated controls. Splenocytes from mice vaccinated intravenously with RALA/*mGPC2* produced highly significant levels of antigen-specific IFN-γ (107.1 SFU/250,000 cells ±18.65; $p < 0.0001$) and IL-2 (100.6 SFU/250,000 cells ±22.61; $p < 0.001$). For both cytokines, this increase in expression was more pronounced than in any other group, particularly in mice vaccinated with the positive control mRNA vaccine and those vaccinated with recombinant GPC2 protein (Figures 3A and 3B). ELISpot also assessed TNF-α expression; however, significant background levels of cytokine were observed across all groups (Figures 3B and S7), skewing the analysis. Additionally, we assessed the potency of the immunization route and established that RALA/*mGPC2* i.v. outperformed i.d. vaccination with ~3-fold higher splenocyte expression of IFN-γ and IL-2.[23]

To further assess the cellular immune response, splenocytes (stimulated or not with GPC2 for 18 h) were stained for T cell surface markers and intracellular IFN-γ, IL-2, and TNF-α. Splenocytes isolated from RALA/*mGPC2*-vaccinated mice showed a trend toward increased TNF-α production by $CD4^+$ and $CD8^+$ T cells, levels higher than those in control groups (Figures 3C and 3D). Similar production of IFN-γ and IL-2 did not show clear trends (Figure S7).

Notably, splenocytes from mice vaccinated with RALA/*mGPC2* had elevated effector (TEM) and central (TCM) memory T cell responses in both $CD4^+$ and $CD8^+$ cells (Figures 3E–3H). The $CD4^+$ population

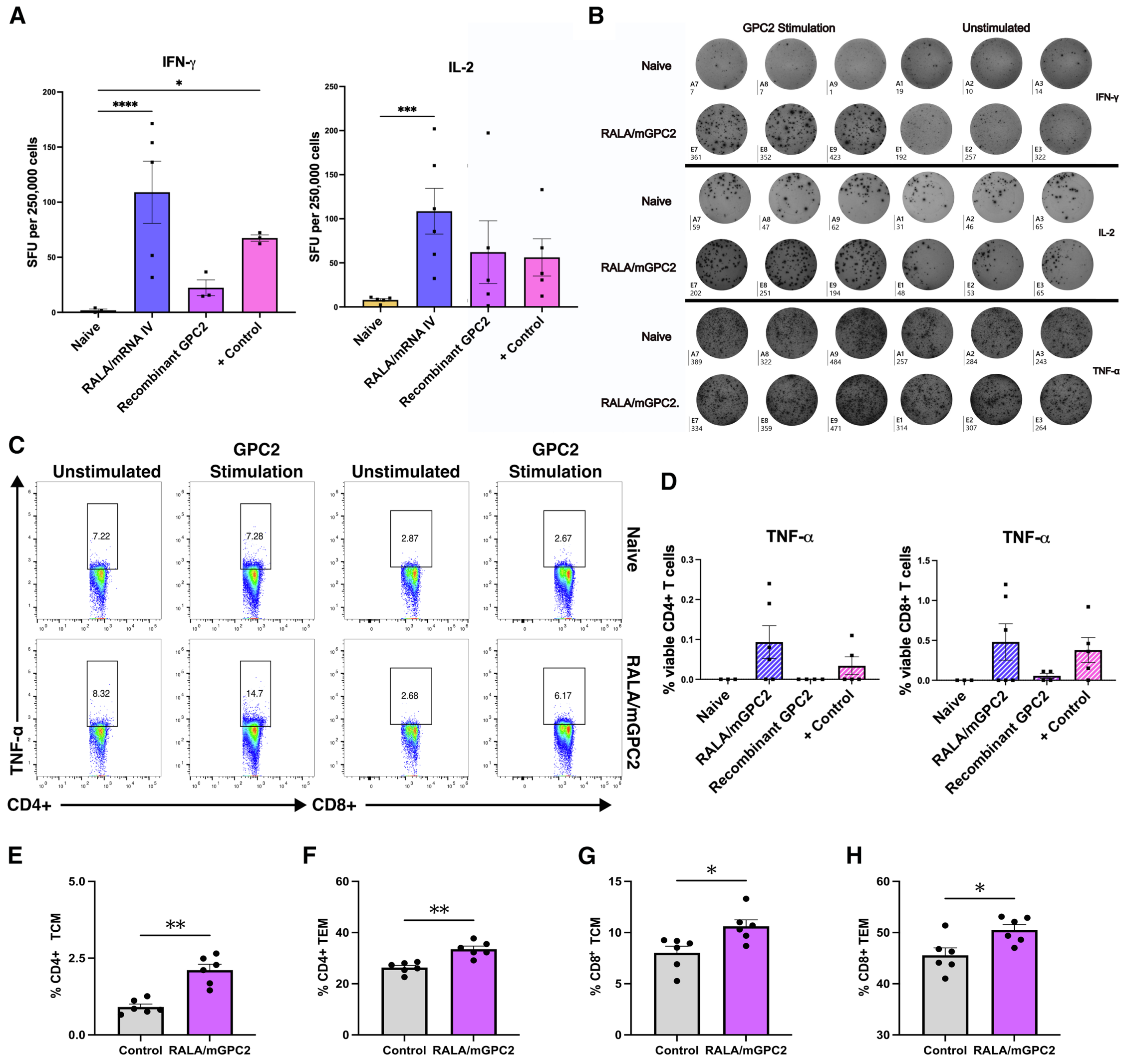

Figure 3. Vaccination with RALA/*mGPC2* stimulates a cellular, antigen-specific immune response *in vivo*

Summary data of ELISpot analysis (A) and representative images for IFN-γ, IL-2, and TNF-α production by splenocytes stimulated with either media alone (unstimulated) or a GPC2 overlapping peptide mix (GPC2 stimulated) *ex vivo* (B). Splenocytes were processed for surface staining ($CD4^+$, $CD8^+$, $CD44^+$, $CD62L^+$) and subsequent flow cytometry (FC) analysis. Representative gating of $CD4^+TNF\text{-}\alpha^+$ and $CD8^+TNF\text{-}\alpha^+$ cells from naive and vaccinated mice is shown in (C), and summary data are graphed in (D). $CD4^+$ and $CD8^+$ central memory (TCM) and effector memory (TEM) subsets were assessed (E–H). Data represent the mean ± SEM ($n = 6$) and were statistically evaluated by an ordinary one-way ANOVA with Tukey's multiple comparisons (A–D) and the Mann-Whitney test (E–H), (*$p \leq 0.05$; **$p \leq 0.01$; ***$p \leq 0.001$; ****$p \leq 0.0001$). Only statistically significant comparisons are graphed.

demonstrated significant increases in $CD4^+$ TCM (2.11 ± 0.45%; p = 0.015, Figure 3E) and $CD4^+$ TEM (33.48 ± 2.97%; p = 0.002, Figure 3F) populations. The $CD8^+$ population demonstrated significant increases in $CD8^+$ TCM (10.61 ± 1.55%; p = 0.015, Figure 3G) $CD8^+$ TEM (50.52 ± 2.54%; p = 0.015, Figure 3H) populations. Thus, our data demonstrated a robust cytotoxic immune response following RALA/*mGPC2* immunization.

The data shown in Figure 3 provide strong rationale that RALA/*mGPC2* stimulates an antigen-specific, cellular immune response to GPC2 in mice.

RALA/*mGPC2* immunization delays tumor uptake and slows tumor progression

Following tumor implantation, the mice were vaccinated with a 20 μg RALA/*mGPC2* regimen on days 11, 15, and 18 (Figure 4A). The mice were monitored three times weekly for the formation of palpable tumors and overall weight (Figure 4B). All the animals maintained similar body weights throughout the study. Compared with the control, immunization with RALA/*mGPC2* delayed tumor development by 10–11 days and significantly lowered the tumor burden (Figure 4C). The RALA/*mGPC2* vaccine was designed against human GPC2. However, the amino acid sequence of human

GPC2 was highly similar to that of mouse GPC2 and shared predicted MHC class I and II epitopes (Figure 4D). Thus, at 60 days post-challenge, the control mice had a median tumor volume of 408.45 mm^3. In contrast, the immunized mice had a median tumor volume of 132.3 mm^3 (Figure 4E) and higher survival rates (Figure S8). Thus, the tumor volume of vaccinated animals was reduced by 70% at this time point, with the potential further to reduce tumor progression via prolonged administration of this vaccine. Additionally, we observed no differences in circulating toxicity biomarkers, namely creatinine, alkaline phosphatase, lactate dehydrogenase, and creatine kinase, which can detect damage to liver, kidney, lung, bone, muscle, brain, or heart tissues, between unvaccinated and RALA/*mGPC2*-vaccinated mice (Figures 4F–4I). Notably, the levels of circulating biomarkers were consistent with the reference intervals for clinical chemistry parameters found in healthy C57BL/6 mice.[24–26]

DISCUSSION

Our work demonstrates, for the first time, that *mGPC2* encapsulated in RALA elicits an antigen-specific cellular immune response, stimulating the generation of both effector and central memory T cells. Additionally, RALA/*mGPC2* vaccination slowed tumor growth in a murine model of *MYCN*-amplified neuroblastoma using the TH-MYCN transgenic mouse-derived 9464D neuroblastoma cell line on a C57BL/6 background.[27] While this cell line represents *MYCN*-amplified neuroblastoma, GPC2 is also expressed in non-MYCN-amplified settings, making the selected model applicable to both genomic landscapes. The ability of the RALA peptide to form nanoscale complexes with DNA and RNA, which are stable at RT, prevent its cargo degradation, and increase transfection efficacy *in vitro* and *in vivo* has been well documented (see Table S1).

RALA is a cationic amphipathic cell-penetrating peptide (CPP) composed of a repeating Arg-Ala-Leu-Ala subunit. The hydrophilic arginine and hydrophobic leucine residues are located on opposite sides of an alpha helix structure, with both regions contributing to major functions of the RALA peptide. Arginine (R), which facilitates nucleic acid binding, and leucine (L), which interacts with lipid membranes, are separated by alanine (A)- rich regions, giving the peptide its amphipathic nature. Additional amino acids, tryptophan (W) and glutamic acid residues at each terminus, serve as spectroscopic probes and enhancers of water solubility at physiological pH, respectively. RALA delivers cargo to cells, with the main mode of internalization being clathrin-mediated endocytosis.[20] NPs in the endosomal environment, with reduced pH, become protonated, increasing the a-helicity of RALA and facilitating endosomal escape and cargo release. Here, the complexation of *mGPC2* with RALA also yielded NPs with favorable physicochemical properties for cellular uptake. Based on size, charge, PDI, and encapsulation efficiency, an N:P ratio of 9 proved most successful, using predefined standards based on previous data generated with the RALA delivery system.[11] Lyophilized particles retained the highly satisfactory characteristics of those prepared and analyzed fresh, indicating that freeze-drying did not significantly affect hydrodynamic size or zeta potential. As expected, western blot analysis confirmed RALA/*mGPC2*-driven expression of human GPC2 on the surface of the mouse DC 2.4 cells, peaking at 24 h. Following RALA/*mGPC2* delivery, the membrane display of the human GPC2, endogenously expressed on the cell surface, confirms mRNA functionality and ensures MHC class I antigen processing, thereby promoting the activation of $CD8^+$ cytotoxic T lymphocyte (CTL) responses. In addition, dendritic cells (DCs) that take up the mRNA or express the antigen can present GPC2 via both MHC class I and II pathways, further supporting $CD8^+$ T cell activation and potentially providing supplementary $CD4^+$ T cell help. This dual mechanism likely contributes to the robust cellular immune responses observed.

Unlike vaccines for infectious diseases, the aim of cancer vaccines is predominantly to stimulate an antigen-specific cellular immune response to eliminate tumor cells. Therefore, we selected the C57BL/6 strain, the most widely chosen strain to study cellular immunity, because of the predominance of Th1-type immunity over the BALB/c mouse strain, which is commonly used to investigate humoral immunity based on the Th2-skewed response.[28,29] The difference here lies at the H2 site of the MHC-I gene locus: C57BL/6 mice show H2b, and BALB/c mice show H2d.

Having established the functionality of the RALA/*mGPC2* vaccine to encode full-length GPC2 *in vitro*, we next evaluated vaccination-induced activation of an antigen-specific cellular response *in vivo*. Central to the induction of a potent anti-tumor response is the generation of tumor-specific CTLs, which can directly lyse cancer cells. CTL responses are also heavily dependent on $CD4^+$ T helper cells; thus, anti-tumor immunity requires the simultaneous expansion of an antigen-specific Th1 $CD4^+$ T cell population to complement cytotoxic $CD8^+$ T cells.[10,30,31] IFN-γ, TNF-α, and IL-2 are key cytokines that coordinate immune responses during vaccination. IFN-γ enhances cytotoxic T cell and NK cell activity, promotes antigen presentation, and supports T cell maturation. TNF-α contributes to tumor cell destruction by activating cytotoxic lymphocytes, macrophages, and antigen-presenting cells within the tumor microenvironment. IL-2 bridges innate and adaptive immunity by activating signaling pathways that promote cellular proliferation and by stimulating T and NK cells. Together, IFN-γ, TNF-α, and IL-2 represent a core cytokine set commonly used to assess polyfunctional T cell responses, tumor cell killing, and vaccine-induced immunity.[10,32,33] Vaccination with RALA/*mGPC2* significantly increased antigen-specific expression of IFN-γ and IL-2, with this vaccine outperforming a previously established in-house positive control vaccine and mice vaccinated with the recombinant GPC2 protein. Despite a lack of conclusive TNF-α ELISpot data, intracellular cytokine staining confirmed TNF-α expression in $CD4^+$ and $CD8^+$ cells post-vaccination. Differences in expression between these two assays could likely be due to timing (18 h versus 48 h), given kinetic differences between expression and release of IFN-γ and TNF-α during an immune response.[34,35] Follow-up studies would benefit from further evaluation of cytokine release kinetics and, indeed, in-depth immune cell profiling in both *MYCN*-amplified and non-amplified scenarios. However, as a first-in-mouse proof-of-concept study, these data

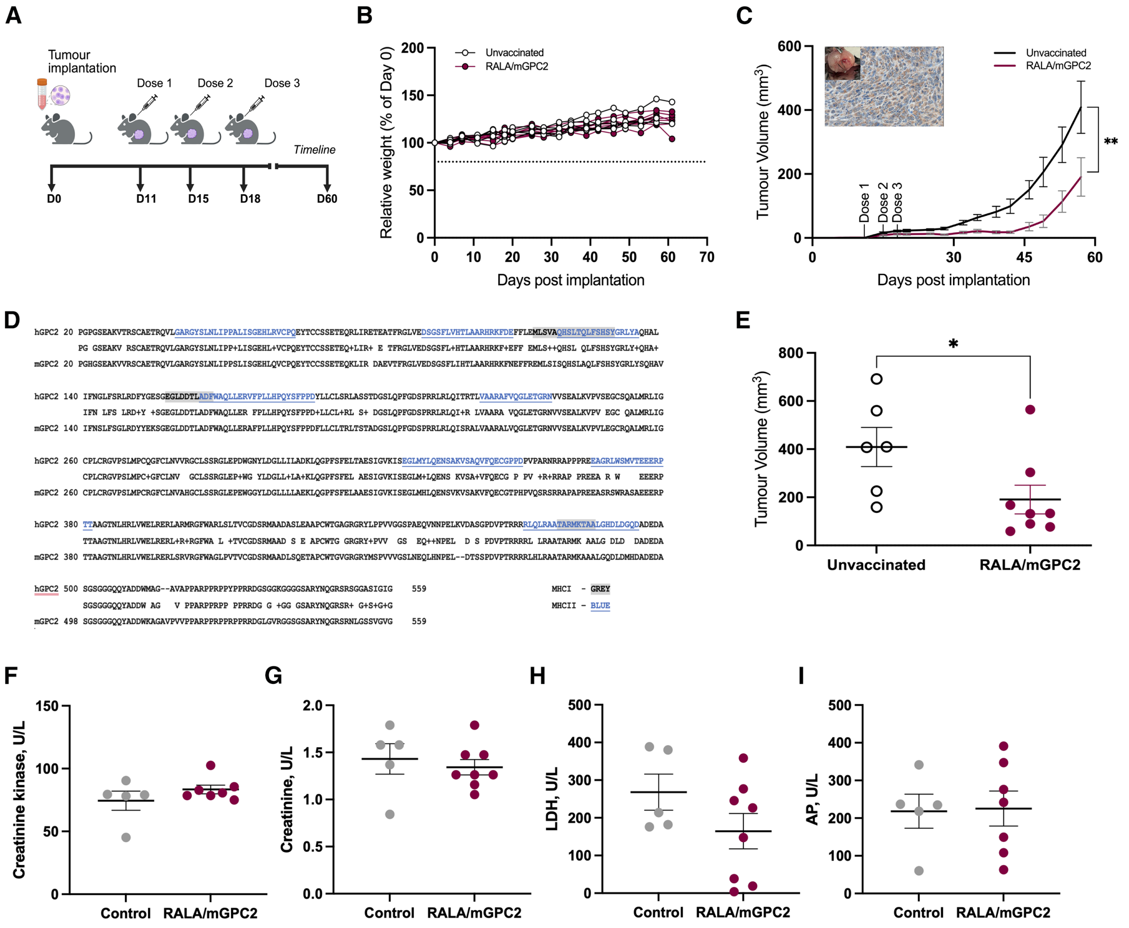

Figure 4. RALA/*mGPC2* vaccination delays the progression of neuroblastoma

(A) Schematic of the immunization schedule (IV). (B) Maintenance of body weight in mice throughout the study. (C) Time to tumor formation in immunized mice; subcutaneous 9464D mouse neuroblastomas were PPFA embedded, followed by IHC staining with an anti-human GPC2 antibody (Bioss, bs-13450r). (D) BLAST of human (NP_689955.1) and murine (NP_766000.1) GPC2 aa sequences followed by epitope prediction. (E) Average tumor volume of control and immunized mice on day 60. Assessment of the RALA/*mGPC2* vaccination safety using serum biomarkers of toxicity: (F) creatinine kinase, CK, (G) creatinine, (H) alkaline phosphatase, AP, (I) lactate dehydrogenase, LDH. The data represent the means ± SEMs and were statistically evaluated by one-way ANOVA (C) and the Mann-Whitney test (E–I), with significance at $p < 0.05$. (*$p < 0.05$, **$p < 0.01$). Illustrations were created in BioRender.com.

provided clear evidence of a GPC2-specific cellular immune response to RALA/*mGPC2* vaccination.

Having confirmed the RALA/*mGPC2*-mediated activation of an antigen-specific T cell response, we next evaluated vaccine-driven tumor control in a C57BL/6 model. Previous RALA-based vaccines demonstrated increased survival of tumor-bearing mice in cervical and prostate cancer models.[12,17,19,36] Here, immunization with RALA/*mGPC2* NPs significantly delayed tumor growth by 11 days, reduced tumor volume by 70%, and prolonged survival. While it would be ideal to achieve a longer delay in tumor growth, or even to eliminate it, a different immunization protocol may be necessary. For example, Johnson et al. (2007) reported that as many as 6 i.d. immunizations with a plasmid expressing rat PAP (pTVG-RP) were necessary to generate an anti-PAP response in Lewis rats.[37]

Translating therapies from the bench to the bedside requires robust preclinical research and animal models that accurately represent the actual disease state. Bosse et al. validated murine models for the preclinical testing of GPC2-directed therapeutics based on 80% sequence homology between mouse and human GPC2 and cell-surface expression. They showed that both mouse and human GPC2-expressing cells were equally susceptible to antibody-drug conjugate-mediated killing.[7] Our work provides a strong rationale for preclinical testing of novel GPC2 vaccines in these models. Given the interspecies sequence homology, most on-target toxicity would be expected to be apparent in mice vaccinated against GPC2, which was not observed in our study. These findings suggest that murine models provide a viable and clinically relevant preclinical setting for evaluating GPC2-directed therapeutics intended for human use. Raman et al. used two genetically unique mouse models: one with *MYCN*

amplification, wild-type *ALK*, and wild-type *TP53*, and the other with no *MYCN* amplification, wild-type *ALK*, and mutated *TP53*. They showed equal susceptibility to their GPC2-ADC.[38] The fact that both models with genetically diverse backgrounds were equally susceptible to GPC2-directed therapy suggests that GPC2 expression was similar between the two. This is especially important when considering the genetically diverse neuroblastoma patient population and ensuring inclusivity in the design of novel therapeutics.

In the context of neuroblastoma, MHC class I (HLA-I) loss or downregulation is very common, especially in high-risk disease. Still, it is heterogeneous and often reversible rather than genetically fixed.[39,40] This attribute should be considered to achieve robust vaccine efficacy. Most often, the loss of MHC expression on tumor cells is mediated by epigenetic events and by transcriptional downregulation of the MHC locus and/or the antigen-processing machinery.[41] For example, DNA hypermethylation and histone acetylation can be reversed by administration of demethylating chemotherapy and HDAC inhibitors. HDAC is increased MHC-I expression in a panel of *in vitro* NBL models[42] and murine 9464D NBL cells.[43] Therefore, the next preclinical vaccine efficacy study would benefit from testing in combination with a therapy such as Vorinostat, an HDAC inhibitor included in neuroblastoma clinical trials.[1]

GPC2 is located at chromosome 7q22.1 and is somatically gained in ~40% of neuroblastomas. Bosse et al. reported significant upregulation of *GPC2* expression with 7q gain (approximately 2- and 1.5-fold, respectively, via both RNA sequencing (n = 64) and mRNA microarrays (n = 118) from the TARGET dataset (accession number: phs000218).[7] This increase was present even in the absence of *MYCN* amplification, a proven driver of *GPC2* expression. Therefore, neuroblastoma patients with a 7q gain may be the primary beneficiaries of this personalized therapeutic approach. While this study focused on neuroblastoma, *GPC2* is upregulated in multiple cancer subtypes, making the RALA/*mGPC2* vaccine an attractive immunotherapy beyond its original application.

mRNA vaccines offer a versatile approach to cancer immunotherapy, with a notable capacity to encode multiple TAAs or epitopes within a single mRNA construct. This platform enables the simultaneous expression of diverse immunogenic peptides, costimulatory molecules, and therapeutic antibodies via an economical, stable delivery system, offering significant adaptability for clinical translation.

Conclusion

This study provides the first preclinical evidence that mRNA vaccines can induce immune responses against endogenous neuroblastoma antigen 2 (GPC2) and delay tumor progression in syngeneic murine models. RALA/*mGPC2* vaccination generated tumor-specific CTL responses without the need for supplemental adjuvants.

The modular design supports combination with other anticancer therapies, positioning mRNA platforms as promising translational candidates. This study highlights the dual advantages of intrinsic immunogenicity and configurable antigen presentation inherent to mRNA-based systems, providing a foundation for further optimization in oncology applications.

MATERIALS AND METHODS

Reagents

The RALA peptide (Bachem, Switzerland) was produced by solid-phase synthesis and supplied as a lyophilized powder with a purity >96%, as determined by desalting. The 30 amino acid sequence of RALA is N- WEARLARALARALARHLARALARALRACEA-C. Trehalose was purchased from Pfanstiehl, USA (Cat# T-104-4).

All mRNAs were sourced from Trilink Biotechnologies (USA). The GPC2 overlapping peptides were from Genscript. Other reagents include: Fluorescamine (Sigma-Aldrich, UK, cat. no. F9015), bovine serum albumin (BSA) (Sigma-Aldrich, UK, cat. no. A3608), Tween 20 (Sigma-Aldrich, UK, cat. no. P1379), Triton X-100 (Sigma-Aldrich, UK, cat. no. *X*100-5 mL), TMB ELISA Substrate (Abcam UK, cat. no. ab171523), Stop solution1x (Abcam, UK, cat. no. ab210900), RNAase (Thermo Fisher Scientific, UK, cat. no. EN0531), UltraPure Water (Thermo Fisher Scientific, UK, cat. no. 10977015), and 4% paraformaldehyde (Fischer Scientific, UK, cat. no. 15424389).

Design and synthesis of the DNA and mRNA

The Human *GPC2* (Clone i.d.: 5240315) cDNA construct was purchased from Horizon Discovery Ltd (#MHS6278-202801883). The full GPC2 protein-coding region was subcloned from the original vector into pcDNA3.1(−) plasmid (InvitrogenTM #V79520) using HindIII (NEB #R0104) and EcoRI (NEB #R0101). DNA sequence was verified using Eurofins Genomics TubeSeq Sanger sequencing and aligned to Human *GPC2* NCBI gene sequence (Gene ID: 221914) using the BLAST alignment tool.

Custom *GPC2* codon-optimized mRNA was ordered from TebuBio covering the full protein-coding region. The modified mRNA transcript was ordered with full substitution of N1-methyl-Pseudo-U, capped (Cap 1) using CleanCap AG, polyadenylated with 120A, DNase I-treated, and silica membrane-purified.

NPs formulation

pGPC2/*mGPC2* was encapsulated with RALA at various N:P ratios. The N:P ratio was calculated based on peptide and nucleic acid mass, considering RALA's positive amino acid content. For an N:P ratio of 1, 1 μg of pDNA required 1.46 μg of RALA, and 1 μg of mRNA required 1.40 μg of RALA, according to the manufacturer's instructions. The plasmid or mRNA was diluted to 0.1 μg/μL using UltraPure DNase/RNase-free distilled water (Invitrogen, #10977015), mixed gently, and combined with RALA (1 μg/μL). After 30 min of incubation at room temperature, NPs formed spontaneously and were immediately used for downstream applications.

Lyophilization of RALA/mRNA NPs

RALA/*pGPC2* and RALA/*mGPC2* NPs were prepared as previously described.[23] Freshly prepared 24% w/v trehalose (Pfanstiehl, USA) in

UltraPure DNase/RNase-free water (Invitrogen) was added to NPs to achieve a final concentration of 12%. The NPs were transferred to 2 mL glass vials, loosely capped, and lyophilized using an SP Scientific AdVantage Pro freeze dryer according to the recipe outlined in Table 1.

Table 1. Lyophilization recipe for RALA/*mGPC2* nanoparticles

Shelf [°C]	ramp [min]	hold [min]	vacuum [mTor]
+5	60	10	600.000
−35	90	0	600.000
−35	0	180	120
−30	60	180	190
−25	60	180	190
+20	120	360	190

NP characterization

NP size, zeta potential, and PDI were measured using a Malvern Zetasizer Nano ZS (Malvern Instruments, UK). Freshly prepared NPs or lyophilized NPs resuspended in UltraPure DNase/RNase-free water were incubated at room temperature for 30 min before analysis. Hydrodynamic size and PDI were measured at 20°C in a microcuvette (Malvern, UK), while zeta potential was determined in a folded capillary zeta cell via Laser Doppler Velocimetry.

NP morphology and size were further characterized by TEM. RALA-encapsulated NPs (0.1 μg/μL) were deposited onto carbon-coated copper grids (TAAB Laboratories, UK) and dried for 24 h. Grids were stained with Uranyless EM stain (EMS, USA) and imaged with a JEOL JEM-1000 TEM at 80 kV.

Encapsulation efficiency and free nucleic acid analysis

Encapsulation efficiency was assessed using ion-exchange chromatography, gel shift assay, and Pico-/RiboGreen fluorescence assays.

Ion-exchange chromatography

Free nucleic acid content was quantified using SP-Sephadex resin (Sigma-Aldrich, GER) hydrated in 1M NaCl and washed with UltraPure DNase/RNase-free water (Invitrogen, #10977015). Nucleic acid or RALA-encapsulated NPs (20 μL; ≥20 mg/mL) were loaded into a packed frit column, eluted with UltraPure water, and analyzed via UV-Vis spectroscopy.

Gel shift assay

Visual assessment of encapsulation efficiency was performed by loading NPs (0.01 μg/μL) mixed with 10*X* Blue Juice loading buffer (Invitrogen, #10816015) onto a 1% SYBR Safe-stained agarose gel (Invitrogen, #S33102). Electrophoresis was conducted at 100 V for 1 h, and the gel was imaged using an Amersham Imager 600.

Pico-/RiboGreen assay

Quantitative fluorescence-based encapsulation efficiency was evaluated using PicoGreen or RiboGreen reagents (Invitrogen, #P11495/#R11491). NPs (0.01 μg/μL) were plated in triplicate in a black 96-well plate with 50 μL of dye solution prepared per the manufacturer's instructions. After 5 min of dark incubation at room temperature, fluorescence was measured (excitation ∼480 nm, emission ∼520 nm) using a Victor3 plate reader. Encapsulation efficiency was calculated by normalizing fluorescence values to free nucleic acid controls.

Cell culture

DC 2.4, mouse bone marrow dendritic cells, were cultured in RPMI medium (Thermo Fisher Scientific, UK, cat. no. 11875093) supplemented with 10% FBS (Thermo Fisher Scientific, UK, cat. no. A5256701), 1% L-glutamine (Thermo Fisher Scientific, UK, cat. no. 25030081), 1*X* MEM NEAA (Thermo Fisher Scientific, UK, cat. no. 10370047), and 25 mM HEPES (Thermo Fisher Scientific, UK, cat. no. 15630056). HEK293, human kidney epithelial cells (ATCC# CRL-1573), were cultured in DMEM medium (Thermo Fisher Scientific, UK, cat. no. 11965092) supplemented with 10% (FBS (Thermo Fisher Scientific, UK, cat. no. A5256701). 9464D is a murine, MYCN-driven neuroblastoma cell line derived from the *TH-MYCN* transgenic mouse model on a C57BL/6 background,[27] a gift from Prof Juliet Gray,[44] and was cultured in RPMI medium (Thermo Fisher Scientific, UK, cat. no. 11875093) supplemented with 10% FBS (Thermo Fisher Scientific, UK, cat. no. A5256701).

Mycoplasma testing was carried out every 30 days on all cell lines in culture using the MycoAlertTM Mycoplasma Detection Kit (Lonza LT07-118).

Cell transfection

HEK293 and DC2.4 cells were seeded in 6-well plates with complete growth media. The next day, the media was replaced with Opti-MEM™ Reduced Serum Medium (Gibco, #31985–070), and cells were incubated at 37°C with 5% CO_2 for 2 h. NPs, freshly prepared or reconstituted from lyophilized stock, were added at 4 μg per well in 200 μL. Plates were gently rotated for even NP distribution and incubated for 5 h at 37°C with 5% CO_2. The media was then replaced with complete growth media, and the cells were incubated until the desired endpoint was reached.

Cell viability assessment

Cell viability was determined using the Cell Titer-Glo 3D assay (Invitrogen, UK). The reagent was thawed overnight at 4°C and then equilibrated to room temperature by placing the bottle in a 22°C water bath for approximately 30 min. Following equilibration, 100 μL of the Cell Titer-Glo 3D reagent was added to each well, and the contents were mixed thoroughly by shaking for 5 min to promote cell lysis or were left to rock for 25 min. Afterward, the plate was incubated at room temperature for an additional 25 min to stabilize the luminescent signal. Subsequently, 100 μL from each well was transferred to an opaque-walled (black) plate. Luminescence was measured using the Victor3 V Plate Reader (PerkinElmer, #1420), and the average luminescence for each condition was calculated using Microsoft Excel. Data were visualized using GraphPad Prism 10.

Protein isolation & western blot

Total protein was isolated from transfected cells by washing with pre-warmed PBS. Cells were lyzed with RIPA buffer (Thermo Fisher Scientific, #89901) containing Halt protease inhibitor cocktail (100*X*) (Thermo Fisher Scientific, #78430) on ice for 30 min. The lysates were centrifuged at 13,200 rpm for 30 min at 4°C, and the supernatants were carefully collected.

Subcellular fractionation of transfected cells in 6-well plates was performed using the Subcellular Protein Fractionation Kit for Cultured Cells (Thermo Fisher Scientific, #78840) according to the manufacturer's protocol. Briefly, buffers were thawed in a water bath at room temperature and kept on ice until needed. To initiate fractionation, 200 μL of ice-cold cytoplasmic extraction buffer (CEB) was added to each well, and the plates were incubated on ice for 10 min with gentle mixing. The resulting lysates were collected into 1.5 mL tubes and centrifuged at 500 × g for 5 min at 4°C. The supernatant, which contained the cytoplasmic fraction, was transferred to pre-chilled tubes. Next, 200 μL of ice-cold membrane extraction buffer (MEB) was added to the cell pellets, followed by vortexing at high speed for 5 s and incubation at 4°C for 10 min with gentle mixing. The samples were centrifuged at 3,000 × *g* for 5 min at 4°C, and the supernatant, representing the membrane fraction, was transferred to pre-chilled tubes.

For nuclear fractionation, 100 μL of ice-cold nuclear extraction buffer (NEB) was added to the remaining cell pellet, vortexed for 15 s at maximum speed, and incubated at 4°C for 30 min with gentle mixing. The samples were then centrifuged at 5,000 × *g* for 5 min at 4°C, and the supernatant (soluble nuclear fraction) was collected. The remaining pellet was treated with 100 μL of room temperature NEB+ buffer, vortexed for 15 s at high speed, and incubated at room temperature for 15 min with gentle vortexing. After a final centrifugation at 16,000 × *g* for 5 min, the supernatant, containing the chromatin-bound nuclear extract, was collected. The two nuclear fractions were then combined.

To isolate the cytoskeleton fraction, 100 μL of room temperature protein extraction buffer (PEB) was added to the remaining cell pellet, vortexed at high speed for 15 s, and incubated at room temperature for 10 min. The samples were centrifuged at 16,000 × *g* for 5 min, and the supernatant (cytoskeleton fraction) was transferred to a fresh tube. Finally, the cytoplasmic and cytoskeleton fractions were combined, and all fractions were stored at −20°C until further protein analysis.

Protein concentrations were quantified using the Pierce BCA Protein Assay Kit (Thermo Fisher Scientific, #23225) according to the manufacturer's protocol. Samples were diluted in 4*X* Bol LDS sample buffer (Invitrogen, #B0007) with the appropriate amount of 10*X* Bolt™ Sample Reducing Agent (Invitrogen, #B0009) and denatured at 95°C for 5 min.

Equal protein amounts (10 μg) were loaded onto Bolt 4%–12% bis-tris plus gels (Invitrogen, #NW04120, #NW04122, #NW04125), and SDS-PAGE was performed at 190 volts for 45 min. Proteins were transferred to nitrocellulose or PVDF membranes at 20 volts for 1 h. The transfer was confirmed by Ponceau S staining (Merck, #6226-79-5), and membranes were blocked with 5% BSA in TBS-T. Primary antibodies: anti-GPC2-HRP (Santa Cruz Biotechnology, #sc-393824, dilution 1:50), anti-GPC2 (Bioss, #bs-13450r, dilution 1:50), anti-VDAC1 (Cell Signaling Technology, #D73D12, dilution 1:1000), a-tubulin (Cell Signaling Technology, #2144, dilution 1:1000), anti-SMAD4 (Cell Signaling Technology, #9515S, dilution 1:1000), anti-GAPDH (Bio-Rad, #MCA4740, dilution 1:1000). Their incubations were performed overnight at 4°C, followed by incubation with HRP-conjugated secondary antibodies (Cell Signaling Technology) at room temperature. Chemiluminescent detection was carried out using the Novex ECL Chemiluminescent Substrate Reagent Kit (Invitrogen, #WP20005), and imaging was performed on an Amersham Imager 600 (GE Healthcare Lifesciences).

Animals and& ethics

8-week-old C57BL/6 female mice (Charles River Laboratories, UK) were used for all *in vivo* studies. The *in vivo* experiments were undertaken at Queen's University Belfast's Biological Services Unit, located in the Medical Biology Center (MBC) building. All experimental protocols complied with the Animal (Scientific Procedures) Act of 1986 and were carried out under project license PPL 2903.

In vivo immune response study

Immunocompetent female C57BL/6 mice were administered a 3-dose vaccine regimen (20 μg) via intravenous injection on days 0, 7, and 11 followed by a 21-days incubation period. On day 32, mice were euthanized via Schedule 1. Cardiac bleeds were taken, and spleens were harvested and processed for subsequent analyses. Due to the novelty of RALA/*mGPC2*, two positive control vaccination groups were included in this study. Mice were vaccinated with recombinant GPC2 (R&D Systems Catalog #: 2304-GP) or a previously verified mRNA vaccine from the McCarthy group. Animals were randomly assigned into 4 groups: naive (n = 5), positive control mRNA vaccine (n = 5), recombinant GPC2 (n = 5), and RALA/mGPC2 (n = 6).

Splenocyte isolation

At the study endpoint, spleens were processed into single-cell suspensions and seeded with the corresponding media master mixes: unstimulated media only, Con A + LPS positive stimulation, and GPC2 overlapping peptides. For intracellular cytokine staining, Brefeldin A 1000*X* (Invitrogen, UK) was diluted 1:5 and 10 μL was added to each well and mixed. Plates were incubated for 6 h in an incubator at 37°C, 5% CO_2. The cells were then collected in an Eppendorf tube and centrifuged at 350 *g* for 5 min at 4°C to pellet cells. Each cell pellet was resuspended in 100 μL of viability stain, then 50 μL of Fc block, and finally 50 μL of an extracellular antibody cocktail (CD4, CD8), incubated on ice for 30 min. After the incubation period, 1 mL of PBS media (PBS +5% FBS +2 mM EDTA) was added, and the mixture was centrifuged at 350 *g* for 5 min at 4°C. 100 μL of IC Fixation buffer (Life Technologies, UK) was added to each well,

and the plate was incubated overnight at 4°C in the dark. The following day, 50 μL of intracellular antibody cocktail (IFN-γ, IL-2, and TNF-α) was added to the samples and incubated at room temperature for 30 min 1 mL of 1*X* permeabilization buffer (Thermo Fisher Scientific, UK) was added to each well, and the samples were centrifuged at 550 *g* for 5 min at room temperature. Samples were resuspended in 500 μL FACS staining buffer (Invitrogen, UK) and analyzed on Attune NxT flow cytometer (Figures S9A and S9B).

ELISpot analysis

ELISpot plates for IFN-γ and IL-2 were prepared as per the manufacturer's instructions. 100 μL of splenocyte cell suspension was added to each well, along with 100 μL of the respective 2*X* master mix, and incubated for 48 h in a humidified incubator at 37°C, 5% CO_2. Post-stimulation ELISpot plates were washed according to the manufacturer's protocol, ensuring the membrane was not touched. Detection antibody (5H4-biotin, provided with the Mabtech kit) was diluted to 1 μg/mL in PBS-0.5% FCS and added at 100 μL/well. Secondary streptavidin-ALP antibodies were diluted (1:1,000) in PBS-0.5% FCS, and 100 μL was added to each well. 100 μL/well of the substrate solution was added to each well and incubated, followed by washing with H_2O. The underdrain tray was removed, and the plates were dried for a minimum of 24 h and analyzed using the Mabtech IRIS ELISpot plate reader (Mabtech Inc., US). The data of technical triplicates were averaged and normalized to unstimulated controls. These values were then plotted as a single data point on each graph. Negative values are set to zero, and outliers have been removed via ROUT analysis (Q = 5%, $N \geq 3$ per group).

Tumor growth kinetics study

The growth kinetics of 9464D mouse neuroblastoma cells in mice were analyzed. Tumors were implanted subcutaneously in immune-competent C57BL/6 mice, under isoflurane anesthesia, in the right flank (total volume of 100 μL PBS cell suspension). Tumor volume, mouse weight, and the general health of the mice were monitored three times weekly. Mice were sacrificed using a schedule 1 method when the tumor volume reached 12 GMD (geometric mean diameter).

In vivo tumor regression study

On day 0, mice were weighed and implanted subcutaneously with $5 \times 10^{\hat{}6}$ 9464D cells in 100 μL PBS under anesthesia (isoflurane, <5%). Animals were randomly assigned to 2 groups: control (unvaccinated; $n = 6$) and RALA/*mGPC2* ($n = 8$). Mice were then immunized with NPs (20 μg) intravenously (100 μL lateral tail vein) in a 3-dose regimen on days 11, 15, and 18 with RALA/*mGPC2* NPs. Tumor volume, mouse weight, and general health were monitored three times weekly. Mice were sacrificed when tumor volume reached a maximum of 12 GMD via schedule 1 methods.

Serum toxicity biomarker analysis

Circulating biomarkers that might indicate toxicity in the heart, liver, kidney, muscles, bone, and brain were assessed in the sera from vaccinated C57BL/6 mice. The following assays were utilized: creatinine kinase (Abcam UK, cat. no. ab155901), creatinine (Assay Genie, cat. no. MOEB2563), lactate dehydrogenase (Assay Genie, cat. no. MOES00965), and alkaline phosphatase (Assay Genie, cat. no. MOFI00633). All assays were performed following the manufacturer's instructions.

Statistical analysis

Statistical analysis was performed using GraphPad Prism 10 (GraphPad Software, USA). To compare (1) the means of two groups, an unpaired two-tailed *t* test with the Mann-Whitney correction, and (2) the means of multiple groups, an ordinary one-way ANOVA with Tukey's multiple comparisons of mean test were performed, with a *p* value output indicating statistical significance. These tests were performed on all data that showed statistical significance, as highlighted, unless otherwise noted in the figure legends. For all data analyses, statistical significance was reported as $p \leq 0.05$, $^*p \leq 0.05$, $^{**}p \leq 0.01$, $^{***}p \leq 0.001$, and $^{****}p \leq 0.0001$.

DATA AND CODE AVAILABILITY

The dataset supporting the conclusions of this article is included within the article and its additional file(s).

ACKNOWLEDGMENTS

O.P. and H.O.M. received support for this project through the HEA North South Program, Health Research Board - The Conor Foley Neuroblastoma Cancer Research Foundation (HRCI-HRB-2022-013). R.S. was funded by the Irish Research Council Postgraduate Program (GOIPG/2023/3540). The funders had no role in the study design, data collection, and analysis, decision to publish, or preparation of the manuscript. This article does not contain any studies with humans performed by any of the authors. Procedures involving the use of animals were performed at Queen's University Belfast's Biological Services Unit, located in the Medical Biology Center (MBC) building. All experimental protocols complied with the Animal (Scientific Procedures) Act of 1986 and were carried out under project license PPL 2903.

AUTHOR CONTRIBUTIONS

E.K., writing – original draft, methodology, investigation, formal analysis, data curation, validation, visualization, and writing – review and editing; C.S., methodology, investigation, formal analysis, visualization, and writing – review and editing; R.S., methodology, investigation, formal analysis, and validation; B.J., methodology, investigation, and formal analysis; E.O.D., methodology, investigation, and formal analysis; F.C., methodology, investigation, and formal analysis; H.O.M., resources, supervision, methodology, funding acquisition, formal analysis, project administration, Conceptualization, and writing – review and editing; O.P., writing – original draft, methodology, data curation, Visualization, supervision, funding acquisition, formal analysis, project administration, conceptualization, and writing – review and editing.

DECLARATION OF INTERESTS

The authors declare that they have no competing interests.

SUPPLEMENTAL INFORMATION

Supplemental information can be found online at https://doi.org/10.1016/j.omton.2026.201244.

REFERENCES

1. DuBois, S.G., Moreno, L., Anderson, J., Asgharzadeh, S., Bagatell, R., Beck-Popovic, M., Belle, J., Berlanga, P., Bird, N.J., Chesler, L., et al. (2025). Accelerating Drug Development for Neuroblastoma: Consensus Statement From the Third Neuroblastoma Drug Development Strategy Forum. Pediatr. Blood Cancer *72*, e31831. https://doi.org/10.1002/pbc.31831.

2. King, E., Struck, R., and Piskareva, O. (2025). The triad in current neuroblastoma challenges: Targeting antigens, enhancing effective cytotoxicity and accurate 3D in vitro modelling. Transl. Oncol. *51*, 102176. https://doi.org/10.1016/j.tranon.2024.102176.

3. Svennerholm, L., Boström, K., Fredman, P., Jungbjer, B., Lekman, A., Månsson, J.-E., and Rynmark, B.-M. (1994). Gangliosides and allied glycosphingolipids in human peripheral nerve and spinal cord. Biochim. Biophys. Acta Lipids Lipid. Metabol. *1214*, 115–123. https://doi.org/10.1016/0005-2760(94)90034-5.

4. Blom, T., Lurvink, R., Aleven, L., Mensink, M., Wolfs, T., Dierselhuis, M., van Eijkelenburg, N., Kraal, K., van Noesel, M., van Grotel, M., and Tytgat, G. (2021). Treatment-Related Toxicities During Anti-GD2 Immunotherapy in High-Risk Neuroblastoma Patients. Front. Oncol. *10*, 601076. https://doi.org/10.3389/fonc.2020.601076.

5. Mueller, I., Ehlert, K., Endres, S., Pill, L., Siebert, N., Kietz, S., Brock, P., Garaventa, A., Valteau-Couanet, D., Janzek, E., et al. (2018). Tolerability, response and outcome of high-risk neuroblastoma patients treated with long-term infusion of anti-GD2 antibody ch14.18/CHO. mAbs *10*, 55–61. https://doi.org/10.1080/19420862.2017.1402997.

6. Chen, G., Luo, D., Zhong, N., Li, D., Zheng, J., Liao, H., Li, Z., Lin, X., Chen, Q., Zhang, C., et al. (2022). GPC2 Is a Potential Diagnostic, Immunological, and Prognostic Biomarker in Pan-Cancer. Front. Immunol. *13*, 857308. https://doi.org/10.3389/fimmu.2022.857308.

7. Bosse, K.R., Raman, P., Zhu, Z., Lane, M., Martinez, D., Heitzeneder, S., Rathi, K.S., Kendsersky, N.M., Randall, M., Donovan, L., et al. (2017). Identification of GPC2 as an Oncoprotein and Candidate Immunotherapeutic Target in High-Risk Neuroblastoma. Cancer Cell *32*, 295–309.e12. https://doi.org/10.1016/j.ccell.2017.08.003.

8. Heitzeneder, S., Bosse, K.R., Zhu, Z., Zhelev, D., Majzner, R.G., Radosevich, M.T., Dhingra, S., Sotillo, E., Buongervino, S., Pascual-Pasto, G., et al. (2022). GPC2-CAR T cells tuned for low antigen density mediate potent activity against neuroblastoma without toxicity. Cancer Cell *40*, 53–69.e9. https://doi.org/10.1016/j.ccell.2021.12.005.

9. Miao, L., Zhang, Y., and Huang, L. (2021). mRNA vaccine for cancer immunotherapy. Mol. Cancer *20*, 41. https://doi.org/10.1186/s12943-021-01335-5.

10. Lorentzen, C.L., Haanen, J.B., Met, Ö., and Svane, I.M. (2022). Clinical advances and ongoing trials of mRNA vaccines for cancer treatment. Lancet Oncol. *23*, e450–e458. https://doi.org/10.1016/S1470-2045(22)00372-2.

11. McCarthy, H.O., McCaffrey, J., McCrudden, C.M., Zholobenko, A., Ali, A.A., McBride, J.W., Massey, A.S., Pentlavalli, S., Chen, K.-H., Cole, G., et al. (2014). Development and characterization of self-assembling nanoparticles using a bio-inspired amphipathic peptide for gene delivery. J. Contr. Release *189*, 141–149. https://doi.org/10.1016/j.jconrel.2014.06.048.

12. Cole, G., Ali, A.A., McErlean, E., Mulholland, E.J., Short, A., McCrudden, C.M., McCaffrey, J., Robson, T., Kett, V.L., Coulter, J.A., et al. (2019). DNA vaccination via RALA nanoparticles in a microneedle delivery system induces a potent immune response against the endogenous prostate cancer stem cell antigen. Acta Biomater. *96*, 480–490. https://doi.org/10.1016/j.actbio.2019.07.003.

13. McCrudden, C.M., McBride, J.W., McCaffrey, J., Ali, A.A., Dunne, N.J., Kett, V.L., Coulter, J.A., Robson, T., and McCarthy, H.O. (2017). Systemic RALA/iNOS Nanoparticles: A Potent Gene Therapy for Metastatic Breast Cancer Coupled as a Biomarker of Treatment. Mol. Ther. Nucleic Acids *6*, 249–258. https://doi.org/10.1016/j.omtn.2016.12.010.

14. Saha, C., Elkashif, A., Gilmore, E.J., Jiang, B., Sun, Y., Duary, R.K., Buckley, N., Dunne, N.J., and McCarthy, H.O. (2025). Development of a nano-vaccine for high-grade serous ovarian cancer. Biomater. Sci. *13*, 2908–2924. https://doi.org/10.1039/D4BM01696C.

15. Bennett, R., Yakkundi, A., McKeen, H.D., McClements, L., McKeogh, T.J., McCrudden, C.M., Arthur, K., Robson, T., and McCarthy, H.O. (2015). RALA-mediated delivery of FKBPL nucleic acid therapeutics. Nanomedicine *10*, 2989–3001. https://doi.org/10.2217/nnm.15.115.

16. McCrudden, C.M., Bennie, L., Chambers, P., Wilson, J., Kerr, M., Ziminska, M., Douglas, H., Kuhn, S., Carroll, E., O'Brien, G., et al. (2023). Peptide delivery of a multivalent mRNA SARS-CoV-2 vaccine. J. Control. Release *362*, 536–547. https://doi.org/10.1016/j.jconrel.2023.08.053.

17. Ali, A.A., McCrudden, C.M., McCaffrey, J., McBride, J.W., Cole, G., Dunne, N.J., Robson, T., Kissenpfennig, A., Donnelly, R.F., and McCarthy, H.O. (2017). DNA vaccination for cervical cancer; a novel technology platform of RALA mediated gene delivery via polymeric microneedles. Nanomedicine *13*, 921–932. https://doi.org/10.1016/j.nano.2016.11.019.

18. Udhayakumar, V.K., De Beuckelaer, A., McCaffrey, J., McCrudden, C.M., Kirschman, J.L., Vanover, D., Van Hoecke, L., Roose, K., Deswarte, K., De Geest, B.G., et al. (2017). Arginine-Rich Peptide-Based mRNA Nanocomplexes Efficiently Instigate Cytotoxic T Cell Immunity Dependent on the Amphipathic Organization of the Peptide. Adv. Healthc. Mater. *6*. https://doi.org/10.1002/adhm.201601412.

19. McCrudden, C.M., McBride, J.W., McCaffrey, J., McErlean, E.M., Dunne, N.J., Kett, V.L., Coulter, J.A., Robson, T., and McCarthy, H.O. (2018). Gene therapy with RALA/iNOS composite nanoparticles significantly enhances survival in a model of metastatic prostate cancer. Cancer Nanotechnol. *9*, 5. https://doi.org/10.1186/s12645-018-0040-x.

20. McErlean, E.M., McCrudden, C.M., McBride, J.W., Cole, G., Kett, V.L., Robson, T., Dunne, N.J., and McCarthy, H.O. (2021). Rational design and characterisation of an amphipathic cell penetrating peptide for non-viral gene delivery. Int. J. Pharm. X. *596*, 120223. https://doi.org/10.1016/j.ijpharm.2021.120223.

21. Souza, T.G.F., Ciminelli, V.S.T., and Mohallem, N.D.S. (2016). A comparison of TEM and DLS methods to characterize size distribution of ceramic nanoparticles. J. Phys, Conf. Ser. *733*, 012039. https://doi.org/10.1088/1742-6596/733/1/012039.

22. Vandebriel, R.J., and Hoefnagel, M.H.N. (2012). Dendritic cell-based in vitro assays for vaccine immunogenicity. Hum. Vaccin. Immunother. *8*, 1323–1325. https://doi.org/10.4161/hv.21350.

23. King, E. (2024). DNA and mRNA Vaccines for Neuroblastoma: Design, Development, and Immunogenicity Testing. PhD Thesis (RCSI University of Medicine and Health Sciences).

24. Rodrigues, W.F., Miguel, C.B., Napimoga, M.H., Oliveira, C.J.F., and Lazo-Chica, J.E. (2014). Establishing Standards for Studying Renal Function in Mice through Measurements of Body Size-Adjusted Creatinine and Urea Levels. Biomed Res. Int. *2014*, 872827. https://doi.org/10.1155/2014/872827.

25. Silva-Santana, G., Bax, J.C., Fernandes, D.C.S., Bacellar, D.T.L., Hooper, C., Dias, A.A.S.O., Silva, C.B., de Souza, A.M., Ramos, S., Santos, R.A., et al. (2020). Clinical hematological and biochemical parameters in Swiss, BALB/c, C57BL/6 and B6D2F1 *Mus musculus*. Animal Model. Exp. Med. *3*, 304–315. https://doi.org/10.1002/ame2.12139.

26. Stahl, F.R., Jung, R., Jazbutyte, V., Ostermann, E., Tödter, S., Brixel, R., Kemmer, A., Halle, S., Rose-John, S., Messerle, M., et al. (2018). Laboratory diagnostics of murine blood for detection of mouse cytomegalovirus (MCMV)-induced hepatitis. Sci. Rep. *8*, 14823. https://doi.org/10.1038/s41598-018-33167-7.

27. Kroesen, M., Nierkens, S., Ansems, M., Wassink, M., Orentas, R.J., Boon, L., den Brok, M.H., Hoogerbrugge, P.M., and Adema, G.J. (2014). A transplantable TH-MYCN transgenic tumor model in C57Bl/6 mice for preclinical immunological studies in neuroblastoma. Int. J. Cancer *134*, 1335–1345. https://doi.org/10.1002/ijc.28463.

28. Gorham, J.D., Güler, M.L., Steen, R.G., Mackey, A.J., Daly, M.J., Frederick, K., Dietrich, W.F., and Murphy, K.M. (1996). Genetic mapping of a murine locus controlling development of T helper 1/T helper 2 type responses. Proc. Natl. Acad. Sci. USA *93*, 12467–12472. https://doi.org/10.1073/pnas.93.22.12467.

29. Yagi, J., Arimura, Y., Takatori, H., Nakajima, H., Iwamoto, I., and Uchiyama, T. (2006). Genetic background influences Th cell differentiation by controlling the capacity for IL-2-induced IL-4 production by naive CD4+ T cells. Int. Immunol. *18*, 1681–1690. https://doi.org/10.1093/intimm/dxl102.

30. Verhoeven, B.M., Mei, S., Olsen, T.K., Gustafsson, K., Valind, A., Lindström, A., Gisselsson, D., Fard, S.S., Hagerling, C., Kharchenko, P.V., et al. (2022). The immune cell atlas of human neuroblastoma. Cell Rep. Med. *3*, 100657. https://doi.org/10.1016/j.xcrm.2022.100657.

31. Rojas, L.A., Sethna, Z., Soares, K.C., Olcese, C., Pang, N., Patterson, E., Lihm, J., Ceglia, N., Guasp, P., Chu, A., et al. (2023). Personalized RNA neoantigen vaccines stimulate T cells in pancreatic cancer. Nature *618*, 144–150. https://doi.org/10.1038/s41586-023-06063-y.

32. Sahin, U., Oehm, P., Derhovanessian, E., Jabulowsky, R.A., Vormehr, M., Gold, M., Maurus, D., Schwarck-Kokarakis, D., Kuhn, A.N., Omokoko, T., et al. (2020). An RNA vaccine drives immunity in checkpoint-inhibitor-treated melanoma. Nature *585*, 107–112. https://doi.org/10.1038/s41586-020-2537-9.

33. Kranz, L.M., Diken, M., Haas, H., Kreiter, S., Loquai, C., Reuter, K.C., Meng, M., Fritz, D., Vascotto, F., Hefesha, H., et al. (2016). Systemic RNA delivery to dendritic cells exploits antiviral defence for cancer immunotherapy. Nature *534*, 396–401. https://doi.org/10.1038/nature18300.

34. Thijs, L.G., and Hack, C.E. (1995). Time course of cytokine levels in sepsis. Intensive Care Med. *21*, S258–S263. https://doi.org/10.1007/BF01740764.

35. Cohen, J.I., Hohman, P., Fulton, R., Turk, S.P., Qin, J., Thatcher, K., and Hornung, R.L. (2010). Kinetics of Serum Cytokines after Primary or Repeat Vaccination with the Smallpox Vaccine. J. Infect. Dis. *201*, 1183–1191. https://doi.org/10.1086/651453.

36. Cole, G., Ali, A.A., McCrudden, C.M., McBride, J.W., McCaffrey, J., Robson, T., Kett, V.L., Dunne, N.J., Donnelly, R.F., and McCarthy, H.O. (2018). DNA vaccination for cervical cancer: Strategic optimisation of RALA mediated gene delivery from a biodegradable microneedle system. Eur. J. Pharm. Biopharm. *127*, 288–297. https://doi.org/10.1016/j.ejpb.2018.02.029.

37. Johnson, L.E., Frye, T.P., Chinnasamy, N., Chinnasamy, D., and McNeel, D.G. (2007). Plasmid DNA vaccine encoding prostatic acid phosphatase is effective in eliciting autologous antigen-specific CD8+ T cells. Cancer Immunol. Immunother. *56*, 885–895. https://doi.org/10.1007/s00262-006-0241-8.

38. Raman, S., Buongervino, S.N., Lane, M.V., Zhelev, D.V., Zhu, Z., Cui, H., Martinez, B., Martinez, D., Wang, Y., Upton, K., et al. (2021). A GPC2 antibody-drug conjugate is efficacious against neuroblastoma and small-cell lung cancer via binding a conformational epitope. Cell Rep. Med. *2*, 100344. https://doi.org/10.1016/j.xcrm.2021.100344.

39. Seeger, R.C. (2011). Immunology and immunotherapy of neuroblastoma. Semin. Cancer Biol. *21*, 229–237. https://doi.org/10.1016/j.semcancer.2011.09.012.

40. Sari, G., and Rock, K.L. (2023). Tumor immune evasion through loss of MHC class-I antigen presentation. Curr. Opin. Immunol. *83*, 102329. https://doi.org/10.1016/j.coi.2023.102329.

41. Garcia-Lora, A., Algarra, I., and Garrido, F. (2003). MHC class I antigens, immune surveillance, and tumor immune escape. J. Cell. Physiol. *195*, 346–355. https://doi.org/10.1002/jcp.10290.

42. Cornel, A.M., Dunnebach, E., Hofman, D.A., Das, S., Sengupta, S., van den Ham, F., Wienke, J., Strijker, J.G.M., van den Beemt, D.A.M.H., Essing, A.H.W., et al. (2022). Epigenetic modulation of neuroblastoma enhances T cell and NK cell immunogenicity by inducing a tumor-cell lineage switch. J. Immunother. Cancer *10*, e005002. https://doi.org/10.1136/jitc-2022-005002.

43. Kroesen, M., Büll, C., Gielen, P.R., Brok, I.C., Armandari, I., Wassink, M., Looman, M.W.G., Boon, L., den Brok, M.H., Hoogerbrugge, P.M., and Adema, G.J. (2016). Anti-GD2 mAb and Vorinostat synergize in the treatment of neuroblastoma. Oncoimmunology *5*, e1164919. https://doi.org/10.1080/2162402X.2016.1164919.

44. Webb, E.R., Lanati, S., Wareham, C., Easton, A., Dunn, S.N., Inzhelevskaya, T., Sadler, F.M., James, S., Ashton-Key, M., Cragg, M.S., et al. (2020). Immune characterization of pre-clinical murine models of neuroblastoma. Sci. Rep. *10*, 16695. https://doi.org/10.1038/s41598-020-73695-9.

OMTON, Volume *34*

Supplemental information

mRNA vaccination using peptide nanoparticles triggers a strong immune response against endogenous GPC2 in a murine neuroblastoma model

Ellen King, Chayanika Saha, Rabia Saleem, Binyumeng Jiang, Eve O'Donoghue, Federica Cottone, Helen O. McCarthy, and Olga Piskareva

Table S1. Comparison of RALA-encapsulated NPs

Cargo	N:P ratio	Hydrodynamic size (nm)	Zeta potential (mV)	PdI	Encapsulation Efficiency	Morphology (TEM)	Ref
**mCoV-2**	9	RALA-SME 3.4μg= 29.7 nm<br>RALA-SME 6.7μg = 30.9 nm<br>RALA-SME 26.9μg= 22.1 nm	RALA-SME 3.4μg= 29.7 mV<br>RALA-SME 6.7μg = 30.9 mV<br>RALA-SME 26.9μg = 22.1 mV	RALA-SME 3.4 μg= 0.23<br>RALA-SME 6.7μg = 0.21<br>RALA-SME 26.9μg = 0.23	> 99%	Spherical	[1]
**1) pFKBPL**<br>**2) siFKBPL**	10	55–65 nm	20–25 mV	< 0.6	/	Spherical	[2]
**miR-26a**	8	Fresh nanoparticles 133.9 nm<br><br>Lyophilised nanoparticles 84.77 nm	25/30 mV	Fresh nanoparticles : 0.33<br><br>Lyophilised nanoparticles : 0.276	Fresh nanoparticles: 81.8%<br><br>Lyophilised nanoparticles: 83.1%	Spherical	[3]
**1)P_4-SedU$_2$**<br>**2)P4-SeT2**	RALA/ P_4-SedU$_2$ 2<br>RALA/ P4-SeT2 5	RALA/ P_4-SedU$_2$ 103.3 nM<br>RALA/ P4-SeT2 138.4 nm	RALA/ P_4-SedU$_2$ 18.1 mV<br>RALA/ P4-SeT2 22.6 mV	RALA/ P_4-SedU$_2$ 0.247<br>RALA/ P4-SeT2 0.391	/	Spherical	[4]
**1)pDNA encoding CRISPR machinery and reporter genes**<br>**2)dCas9-VPR**<br>**3)Cas9 protein pre-complexed with gRNAs**	1) 10<br>2) 15<br>3)100x	< 150 nm	20 – 30 mV	≥ 0.2	/	Spherical	[5]

**Alendronate**	10	< 100 nm	16 mV	Fresh nanoparticles: 0.52<br>Lyophilised nanoparticles: 0.34	> 95%	Spherical	6
**E6/E7**	10	150 nm	23 mV	< 0.3	>90%	Spherical	7
**RIS**	1	< 100 nm	Between 10 mV and 30 mV	< 0.2	> 90%	Spherical	8
**iNOS**	10	60 nm	25 mV	/	/	Spherical	9
**α-TCP**	5	43 nm	26 mV	/	/	Spherical	10
**PLA-PEG**	10	70.6±7.5 nm	25 mV	0.240±0.054	> 80%	Spherical	11
**siMMP-9**	9	100–110 nm	37.5 mV to 39.8 mV	< 0.35	/	Spherical	12
**pEGFP-N1**	10	126.1 ± 11.9 nm	18.3 ± 4.1 mV	/	/	Spherical	13
**pEGFP-N1**	10	spray-dried 140 to 307 nm	Spry-dried 18 to 37 mV	/	From 25% to 83%, most > 65%	Spherical	14
**1)pEGFP-N1 2)PLUX 3) HPV-16 E6/E7**	6	Fresh nanoparticles 37.96 nm<br>Lyophilised nanoparticles 74.93 nm	Fresh nanoparticles 17.92 mV<br>lyophilised nanoparticles 11.77 mV	/	80% at N:P 1, decreasing to about 20% at N:P ratios ≥2,	Spherical	15

**pPSCA**	10	47.27 to 93.09	4.53 to 13.57 mV at N:P ratios ≥4, stabilising as the N:P ratio increased.	/	90%	Spherical	16
**pEGFP-N1**	10	70.6 ± 7.5 nm	+34.47 ± 3.19 mV	0.24 ± 0.05	/	Spherical	17
**pCMV-Luc**	10	100 nm	10-30 mV	/	/	Spherical	18
**EGFP-N1**	10	51 nm	29 mV	0.35	>90%	Spherical	19
**EGFP-N1**	10	187.13 nm	20 mV	0.44	90%	Spherical	20
**siFKBPL**	6	76.6 nm	16.5 mV	/	/	Spherical	21
**sFlt-1-e15a**	10	88 nm	30 mV	/	/	Spherical	22
**α-TCP**	5	100 nm	< 30mV	/	/	Spherical	23
**RALA/ mRNA eGFP**	10	91 nm	26.3 mV	/	88%	Spherical	24

Table S2. Critical Quality Attributes of Nanoparticles for Drug Delivery.

Characteristic	***Acceptable***	***Exceptional***	***Pfizer/BioNTech*** [25,26]	***Moderna*** [27]
**Hydrodynamic Size (nm)**	< 200	< 100	80-100	~220
**Zeta Potential (Charge) (mV)**	~ 20	~ 20	Neutral	Neutral

**Polydispersity Index (PdI)**	< 0.4	< 0.2	~0.2	~0.2

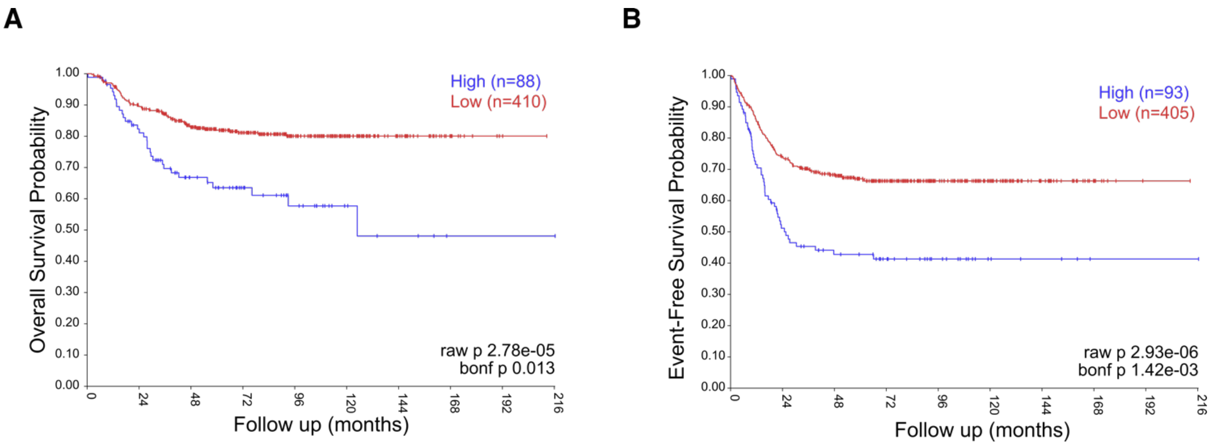

Figure S1. High GPC2 expression correlates with worse overall and event-free survival in a cohort of neuroblastoma patients.
Using Kaplan Meier survival analysis, the significance of high and low GPC2 expression in the SEQC dataset (n=498, [28]) of neuroblastoma patient tumours was assessed based on overall survival (A) and event-free survival (B) in R2: Genomics Analysis and Visualisation Platform (http://r2.amc.nl).

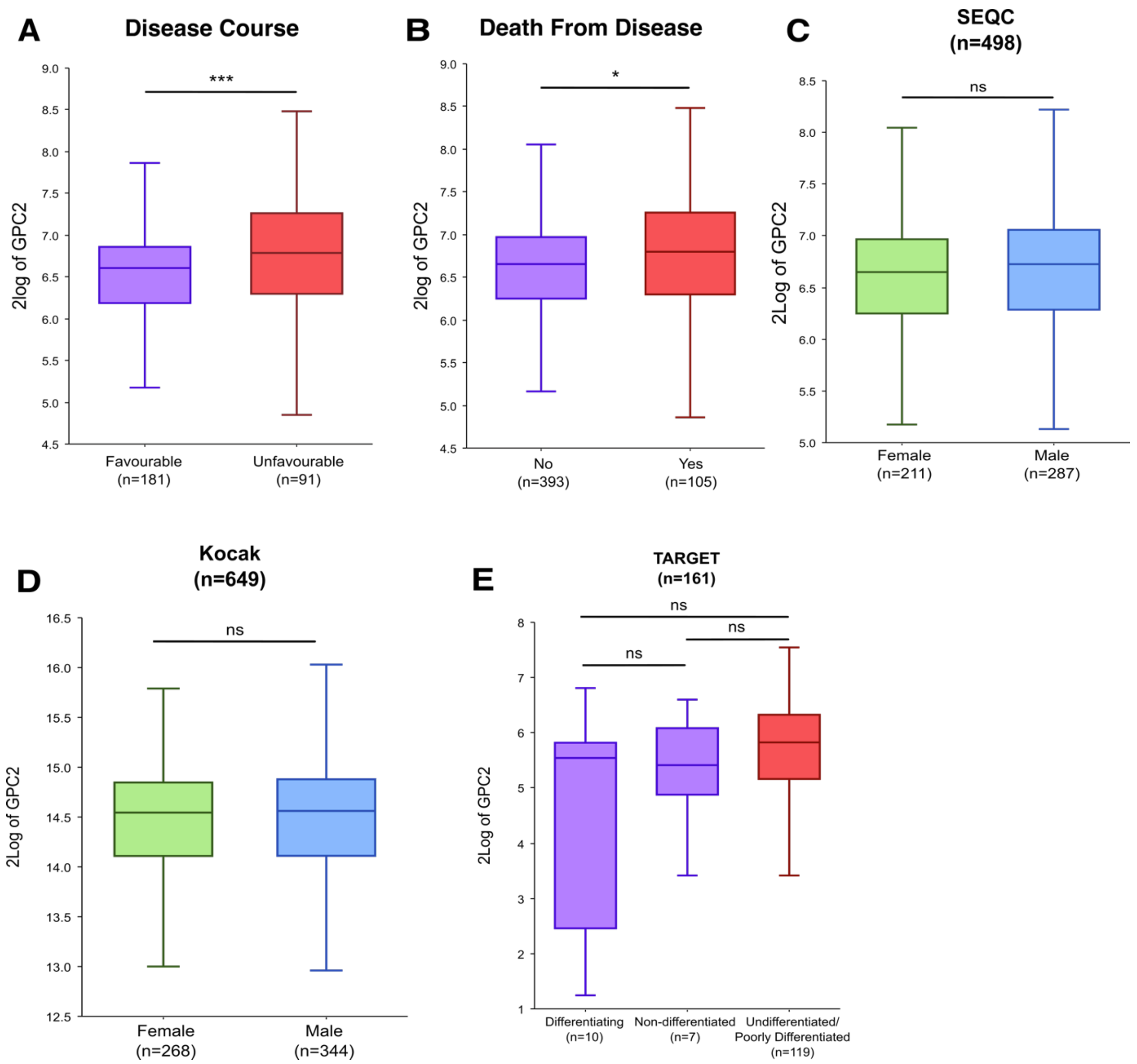

Figure S2. *GPC2* expression analysis vs clinical outcome. (A) disease course and (B) death from disease were examined and correlated with *GPC2* expression. All indicators predictive of adverse clinical outcome, highlighted in red, significantly correlated with increased *GPC2* expression. *GPC2* expression was compared with indicators of clinical outcome using the SEQC dataset (n=498, [28]) in R2: Genomics Analysis and Visualisation Platform (http://r2.amc.nl). Using the (C) SEQC (n=498) and (D) Kocak (n=649) datasets with R2, GPC2 expression was correlated with the gender of the patients, male or female. (E) The association of GPC2 expression with tumour differentiation status was analysed using the TARGET dataset (n=161), with the undifferentiated/poorly differentiated subset, highlighted in red. To determine the significance of GPC2 expression between subsets in each indicator, unpaired t-tests were performed. (*$p \leq 0.05$; **$p \leq 0.01$; ***$p \leq 0.001$; ****$p \leq 0.0001$)

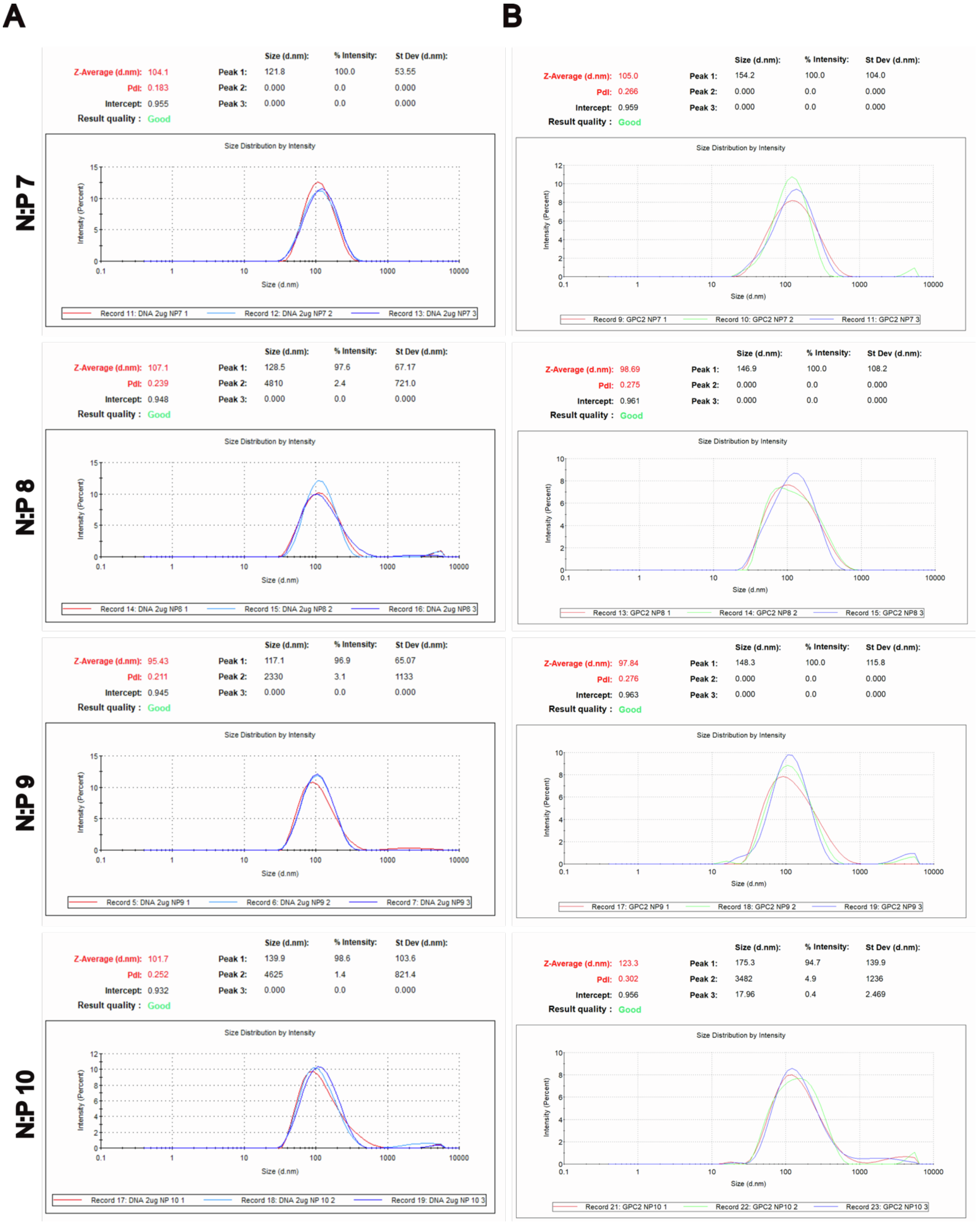

Figure S3. Physicochemical characterisation of RALA/pDNA and RALA/mRNA nanoparticle. Representative size distribution curves of RALA/pGPC2 and RALA/mGPC2 nanoparticles of N:P ratio 7-10. Each curved line represents one repeat out of technical triplicate measurements (N = 3).

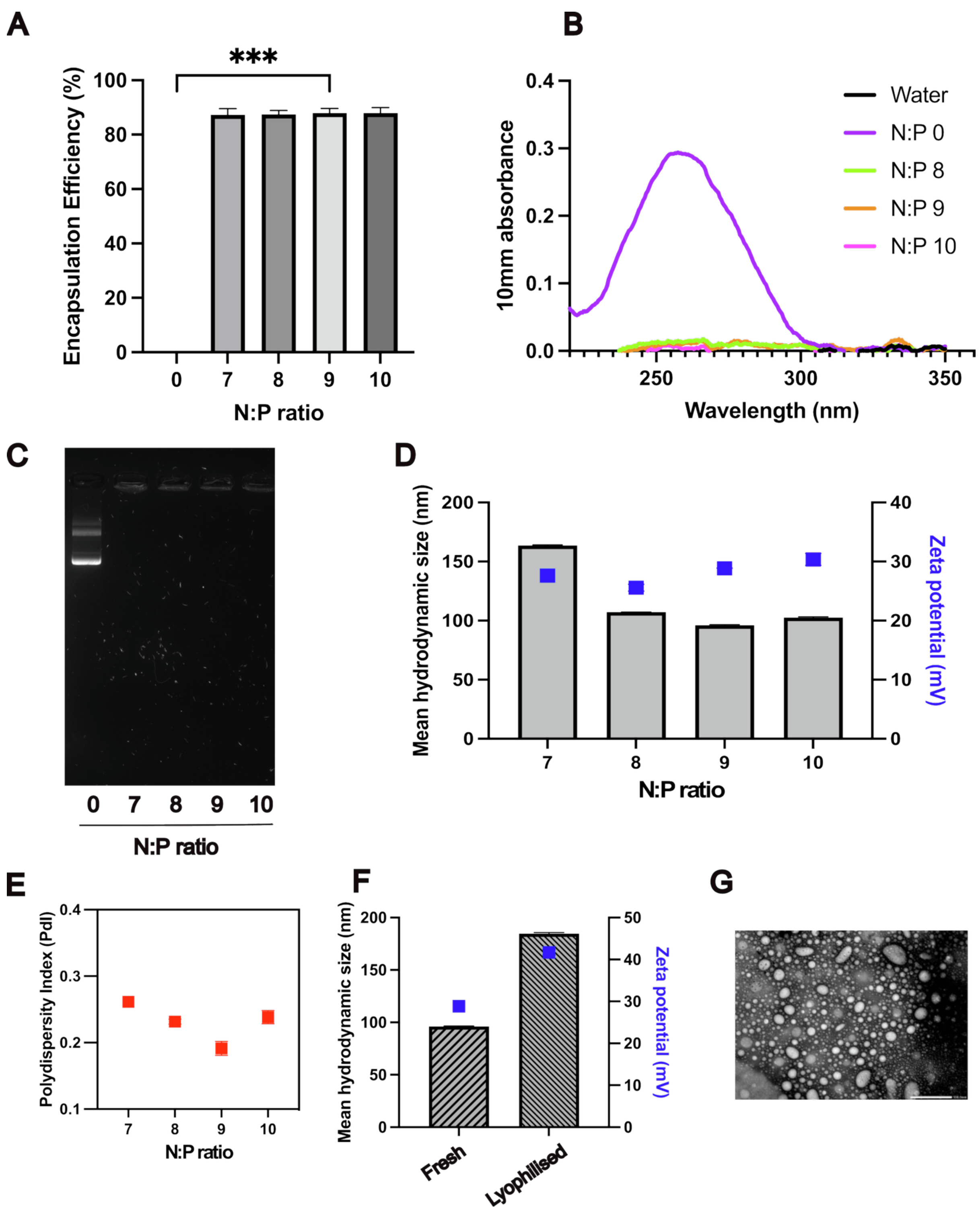

Figure S4. Encapsulation efficiency of RALA/pGPC2 nanoparticles. Encapsulation efficiency of N:P ratios 7-10 was analysed using ion-exchange chromatography (A), PicogreenTM (pGPC2) (B) and agarose gel-shift assay (C). Complexes were incubated at room temperature for 30 min followed by measurement of hydrodynamic size, zeta potential (D) and polydispersity index (E) using a Malvern Zetasizer Nano ZS instrument. Comparison of characteristics of handheld and lyophilised RALA/pGPC2 nanoparticles at N:P ratio 9 (F). Hydrodynamic size, zeta potential and polydispersity index were measured using a Malvern Zetasizer Nano ZS instrument. (N = 3 ± SEM). Statistical analysis was carried out by multiple unpaired T tests, assuming normal distribution. RALA/*pGPC2* NPs were prepared at N:P 9 at a concentration of 1μg/μL and loaded onto carbon-coated copper 400 mesh grids (TAAB Laboratories, UK) (G). Following drying, the samples were stained with 5% uranyl acetate in methanol at room temperature for 1 min, washed with 50% ethanol then molecular grade water

and allowed to dry again. NPs were imaged using a JEM-1400Plus Transmission Electron Microscope (Joel, USA) at an accelerating voltage of 120 kV. Scale bars: 100 nm.

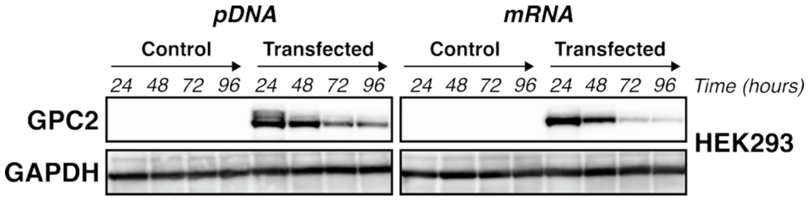

Figure S5. RALA-encapsulated *pGPC2* and *mGPC2* transfection of HEK293 cells at various timepoints.
HEK293 cells were transfected with 1μg of RALA/*pGPC2* and RALA/*mGPC2* NPs for 24, 48, 72 and 96 h. Total protein was isolated and subjected to Western blot analysis to identify GPC2 (~63kDa) overexpression. GAPDH (~36kDa) was used a loading control.

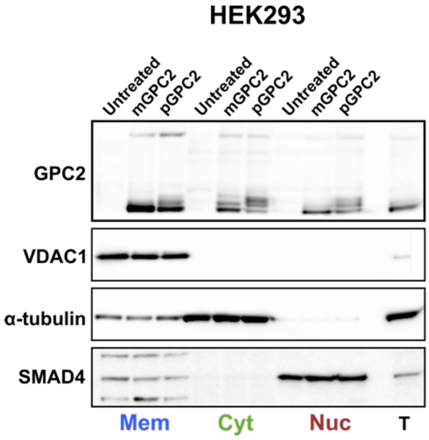

Figure S6. Western blot analysis of HEK293 cells transfected with RALA/pGPC2 and RALA/mGPC2 following subcellular fractionation.
Cells were transfected with 1ug of RALA/mGPC2 and RALA/pGPC2 NPs for 24 and 48 h. Protein from membrane, cytoplasmic and nuclear fractions was isolated and subjected to Western blot analysis to identify GPC2 (~63kDa) overexpression. VDAC1 (30-37kDa), a-tubulin (50kDa) and SMAD4 (65kDa) were used as loading controls for each fraction, respectively.

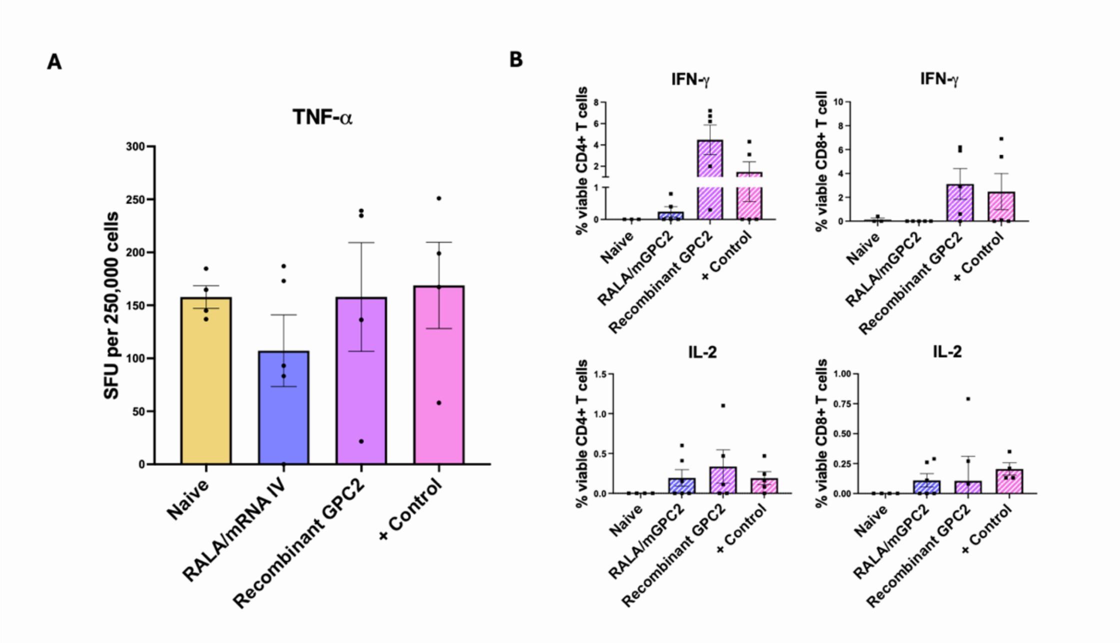

Figure S7. ELISpot analysis of TNF-α, IFN-γ,γ and IL-2 release by splenocytes. ELISpot analysis of cytokine secretion from splenocytes isolated from mice vaccinated with in-house positive control (+ Control), recombinant GPC2, RALA-encapsulated *mGPC2* (RALA/*mGPC2*) compared to unvaccinated mice (Naïve). Vaccinations were delivered i.v. Spot-forming units were counted using Mabtech software. The data shown is an average of technical triplicates and normalized to media-only controls. Negative values are set to zero, and outliers have been removed via ROUT analysis (Q = 5%) using GraphPad PRISM. N ≥ 3 per group. To evaluate statistical differences, an Ordinary one-way ANOVA with Turkey's multiple comparisons was carried out using GraphPad PRISM. Gaussian distribution and standard deviation were assumed based on parametric testing (*$p \leq 0.05$; **$p \leq 0.01$; ***$p \leq 0.001$; ****$p \leq 0.0001$).

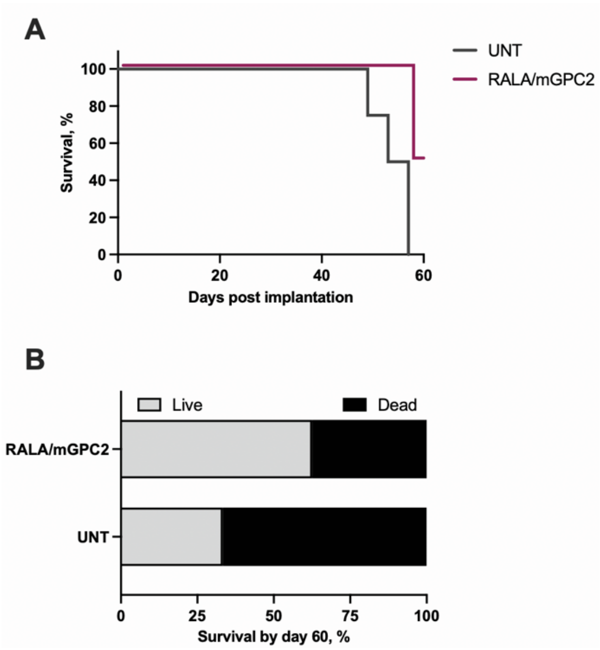

Figure S8. Survival analysis of RALA/mGPC2 vaccinated and unvaccinated mice by day 60. (A) Kaplan-Meier survival analysis; (B) Proportion of live and dead animals.

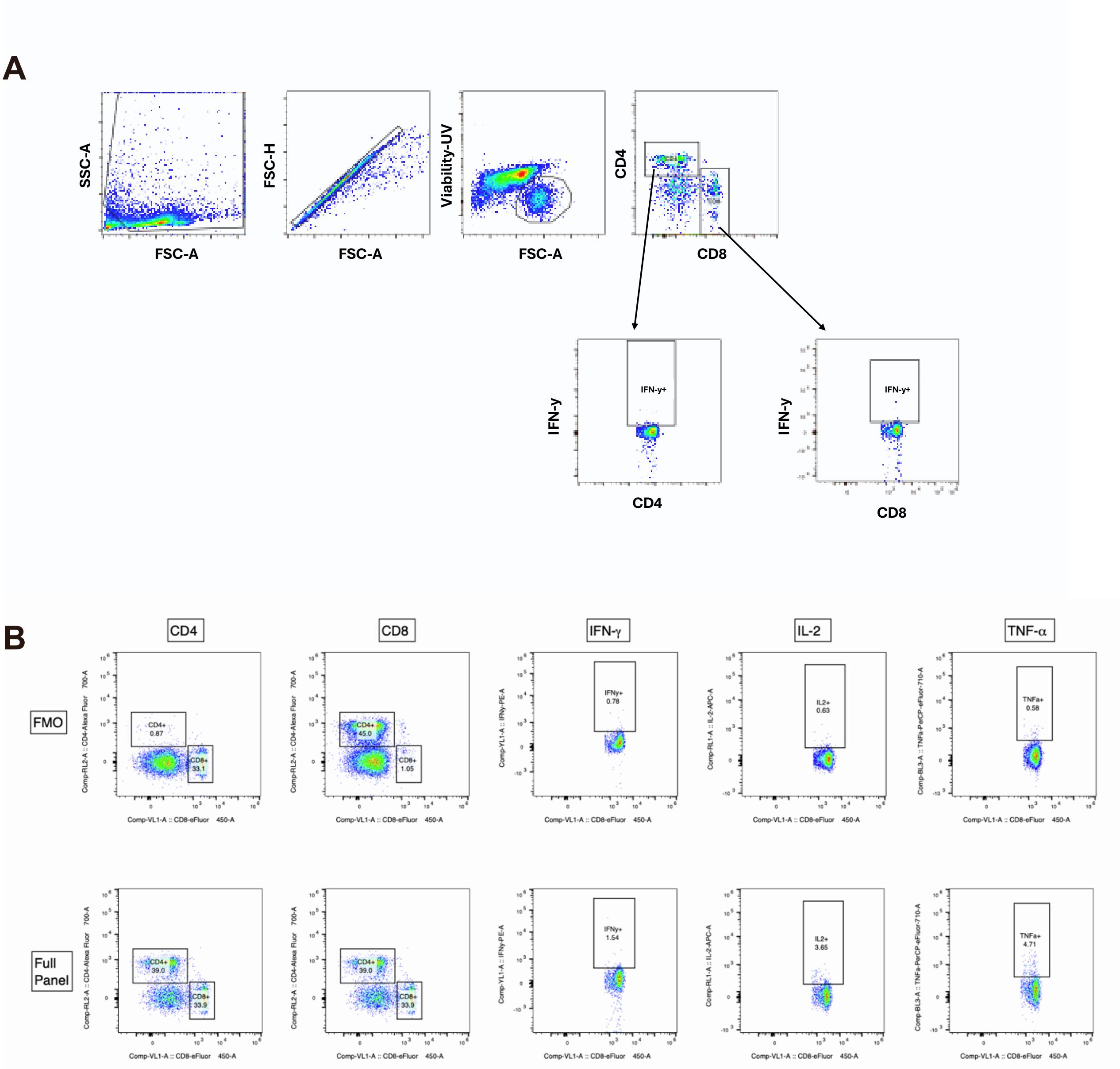

Figure S9. Gating strategy (A) and Fluorescence Minus One (FMO) controls for IFN-γ (B).

References:

1. McCrudden, C.M., Bennie, L., Chambers, P., Wilson, J., Kerr, M., Ziminska, M., Douglas, H., Kuhn, S., Carroll, E., O'Brien, G., et al. (2023). Peptide delivery of a multivalent mRNA SARS-CoV-2 vaccine. Journal of Controlled Release *362*, 536–547. https://doi.org/10.1016/j.jconrel.2023.08.053.
2. Bennett, R., Yakkundi, A., McKeen, H.D., McClements, L., McKeogh, T.J., McCrudden, C.M., Arthur, K., Robson, T., and McCarthy, H.O. (2015). RALA-mediated delivery of FKBPL nucleic acid therapeutics. Nanomedicine *10*, 2989–3001. https://doi.org/10.2217/nnm.15.115.
3. Sadowska, J.M., Ziminska, M., Ferreira, C., Matheson, A., Balouch, A., Bogle, J., Wojda, S., Redmond, J., Elkashif, A., Dunne, N., et al. (2023). Development of miR-26a-activated scaffold to promote healing of critical-sized bone defects through angiogenic and osteogenic mechanisms. Biomaterials *303*, 122398. https://doi.org/10.1016/j.biomaterials.2023.122398.
4. Wilson, J.J., Bennie, L., Eguaogie, O., Elkashif, A., Conlon, P.F., Jena, L., McErlean, E., Buckley, N., Englert, K., Dunne, N.J., et al. (2024). Synthesis and characterisation of a nucleotide based pro-drug formulated with a peptide into a nano-chemotherapy for colorectal cancer. Journal of Controlled Release *369*, 63–74. https://doi.org/10.1016/j.jconrel.2024.03.036.
5. Graham, J.P., Castro, J.G., Werba, L.C., Fardone, L.C., Francis, K.P., Ramamurthi, A., Layden, M., McCarthy, H.O., and Gonzalez-Fernandez, T. (2024). Versatile Cell Penetrating Peptide for Multimodal CRISPR Gene Editing in Primary Stem Cells. Preprint, https://doi.org/10.1101/2024.09.23.614499 https://doi.org/10.1101/2024.09.23.614499.
6. Jena, L.N., Bennie, L.A., McErlean, E.M., Pentlavalli, S., Glass, K., Burrows, J.F., Kett, V.L., Buckley, N.E., Coulter, J.A., Dunne, N.J., et al. (2021). Exploiting the anticancer effects of a nitrogen bisphosphonate nanomedicine for glioblastoma multiforme. J. Nanobiotechnology *19*, 127. https://doi.org/10.1186/s12951-021-00856-x.
7. Ali, A.A., McCrudden, C.M., McCaffrey, J., McBride, J.W., Cole, G., Dunne, N.J., Robson, T., Kissenpfennig, A., Donnelly, R.F., and McCarthy, H.O. (2017). DNA vaccination for cervical cancer; a novel technology platform of RALA mediated gene delivery via polymeric microneedles. Nanomedicine *13*, 921–932. https://doi.org/10.1016/j.nano.2016.11.019.
8. Glass, K., Fines, C., Coulter, P., Jena, L., McCarthy, H.O., and Buckley, N. (2024). Development and Characterization of a Peptide-Bisphosphonate Nanoparticle for the Treatment of Breast Cancer. Mol. Pharm. *21*, 4970–4982. https://doi.org/10.1021/acs.molpharmaceut.4c00299.
9. McCrudden, C.M., McBride, J.W., McCaffrey, J., Ali, A.A., Dunne, N.J., Kett, V.L., Coulter, J.A., Robson, T., and McCarthy, H.O. (2017). Systemic RALA/iNOS Nanoparticles: A Potent Gene Therapy for Metastatic Breast Cancer Coupled as a Biomarker of Treatment. Mol. Ther. Nucleic Acids *6*, 249–258. https://doi.org/10.1016/j.omtn.2016.12.010.
10. O'Doherty, M., Mulholland, E.J., Chambers, P., Pentlavalli, S., Ziminska, M., Chalanqui, M.J., Pauly, H.M., Sathy, B.N., Donahue, T.H., Kelly, D.J., et al. (2020). Improving the Intercellular Uptake and Osteogenic Potency of Calcium Phosphate via Nanocomplexation with the RALA Peptide. Nanomaterials *10*, 2442. https://doi.org/10.3390/nano10122442.
11. Jain, A.K., Massey, A., Yusuf, H., Kett, V.L., McDonald, D., and McCarthy, H. (2015). Development of polymeric–cationic peptide composite nanoparticles, a

nanoparticle-in-nanoparticle system for controlled gene delivery. Int. J. Nanomedicine, 7183. https://doi.org/10.2147/IJN.S95245.
12. Yan, L.-P., Castaño, I.M., Sridharan, R., Kelly, D., Lemoine, M., Cavanagh, B.L., Dunne, N.J., McCarthy, H.O., and O'Brien, F.J. (2020). Collagen/GAG scaffolds activated by RALA-siMMP-9 complexes with potential for improved diabetic foot ulcer healing. Materials Science and Engineering: C *114*, 111022. https://doi.org/10.1016/j.msec.2020.111022.
13. Ziminska, M., Wilson, J.J., McErlean, E., Dunne, N., and McCarthy, H.O. (2020). Synthesis and Evaluation of a Thermoresponsive Degradable Chitosan-Grafted PNIPAAm Hydrogel as a "Smart" Gene Delivery System. Materials *13*, 2530. https://doi.org/10.3390/ma13112530.
14. Munir, M., Kett, V.L., Dunne, N.J., and McCarthy, H.O. (2022). Development of a Spray-Dried Formulation of Peptide-DNA Nanoparticles into a Dry Powder for Pulmonary Delivery Using Factorial Design. Pharm. Res. *39*, 1215–1232. https://doi.org/10.1007/s11095-022-03256-4.
15. Cole, G., Ali, A.A., McCrudden, C.M., McBride, J.W., McCaffrey, J., Robson, T., Kett, V.L., Dunne, N.J., Donnelly, R.F., and McCarthy, H.O. (2018). DNA vaccination for cervical cancer: Strategic optimisation of RALA mediated gene delivery from a biodegradable microneedle system. European Journal of Pharmaceutics and Biopharmaceutics *127*, 288–297. https://doi.org/10.1016/j.ejpb.2018.02.029.
16. Cole, G., Ali, A.A., McErlean, E., Mulholland, E.J., Short, A., McCrudden, C.M., McCaffrey, J., Robson, T., Kett, V.L., Coulter, J.A., et al. (2019). DNA vaccination via RALA nanoparticles in a microneedle delivery system induces a potent immune response against the endogenous prostate cancer stem cell antigen. Acta Biomater. *96*, 480–490. https://doi.org/10.1016/j.actbio.2019.07.003.
17. Jain, A.K., Yusuf, H., Pattani, A., McCarthy, H.O., McDonald, D.M., and Kett, V.L. (2014). Development of a method to quantify the DNA content in cationic peptide–DNA nanoparticles. J. Pharm. Biomed. Anal. *100*, 236–242. https://doi.org/10.1016/j.jpba.2014.07.036.
18. McCaffrey, J., McCrudden, C.M., Ali, A.A., Massey, A.S., McBride, J.W., McCrudden, M.T.C., Vicente-Perez, E.M., Coulter, J.A., Robson, T., Donnelly, R.F., et al. (2016). Transcending epithelial and intracellular biological barriers; a prototype DNA delivery device. Journal of Controlled Release *226*, 238–247. https://doi.org/10.1016/j.jconrel.2016.02.023.
19. McCarthy, H.O., McCaffrey, J., McCrudden, C.M., Zholobenko, A., Ali, A.A., McBride, J.W., Massey, A.S., Pentlavalli, S., Chen, K.-H., Cole, G., et al. (2014). Development and characterization of self-assembling nanoparticles using a bio-inspired amphipathic peptide for gene delivery. Journal of Controlled Release *189*, 141–149. https://doi.org/10.1016/j.jconrel.2014.06.048.
20. McErlean, E.M., McCrudden, C.M., McBride, J.W., Cole, G., Kett, V.L., Robson, T., Dunne, N.J., and McCarthy, H.O. (2021). Rational design and characterisation of an amphipathic cell penetrating peptide for non-viral gene delivery. Int. J. Pharm. *596*, 120223. https://doi.org/10.1016/j.ijpharm.2021.120223.
21. Mulholland, E.J., Ali, A., Robson, T., Dunne, N.J., and McCarthy, H.O. (2019). Delivery of RALA/siFKBPL nanoparticles via electrospun bilayer nanofibres: An innovative angiogenic therapy for wound repair. Journal of Controlled Release *316*, 53–65. https://doi.org/10.1016/j.jconrel.2019.10.050.
22. McNally, R., Alqudah, A., McErlean, E.M., Rennie, C., Morshed, N., Short, A., McGrath, K., Shimoni, O., Robson, T., McCarthy, H.O., et al. (2021). Non-Viral Gene Delivery

Utilizing RALA Modulates sFlt-1 Secretion, Important for Preeclampsia. Nanomedicine *16*, 1999–2012. https://doi.org/10.2217/nnm-2021-0180.

23. Sathy, B.N., Olvera, D., Gonzalez-Fernandez, T., Cunniffe, G.M., Pentlavalli, S., Chambers, P., Jeon, O., Alsberg, E., McCarthy, H.O., Dunne, N., et al. (2017). RALA complexed α-TCP nanoparticle delivery to mesenchymal stem cells induces bone formation in tissue engineered constructs in vitro and in vivo. J. Mater. Chem. B *5*, 1753–1764. https://doi.org/10.1039/C6TB02881K.
24. Udhayakumar, V.K., De Beuckelaer, A., McCaffrey, J., McCrudden, C.M., Kirschman, J.L., Vanover, D., Van Hoecke, L., Roose, K., Deswarte, K., De Geest, B.G., et al. (2017). Arginine-Rich Peptide-Based mRNA Nanocomplexes Efficiently Instigate Cytotoxic T Cell Immunity Dependent on the Amphipathic Organization of the Peptide. Adv. Healthc. Mater. *6*. https://doi.org/10.1002/adhm.201601412.
25. Pardi, N., Tuyishime, S., Muramatsu, H., Kariko, K., Mui, B.L., Tam, Y.K., Madden, T.D., Hope, M.J., and Weissman, D. (2015). Expression kinetics of nucleoside-modified mRNA delivered in lipid nanoparticles to mice by various routes. Journal of Controlled Release *217*, 345–351. https://doi.org/10.1016/j.jconrel.2015.08.007.
26. Kudsiova, L., Lansley, A., Scutt, G., Allen, M., Bowler, L., Williams, S., Lippett, S., Stafford, S., Tarzi, M., Cross, M., et al. (2021). Stability testing of the Pfizer-BioNTech BNT162b2 COVID-19 vaccine: A translational study in UK vaccination centres. BMJ Open Science *5*. https://doi.org/10.1136/bmjos-2021-100203.
27. Hermosilla, J., Alonso-García, A., Salmerón-García, A., Cabeza-Barrera, J., Medina-Castillo, A.L., Pérez-Robles, R., and Navas, N. (2023). Analysing the In-Use Stability of mRNA-LNP COVID-19 Vaccines Comirnaty™ (Pfizer) and Spikevax™ (Moderna): A Comparative Study of the Particulate. Vaccines (Basel). *11*. https://doi.org/10.3390/vaccines11111635.
28. Su, Z., Fang, H., Hong, H., Shi, L., Zhang, W., Zhang, W., Zhang, Y., Dong, Z., Lancashire, L.J., Bessarabova, M., et al. (2014). An investigation of biomarkers derived from legacy microarray data for their utility in the RNA-seq era. Genome Biol. *15*, 523. https://doi.org/10.1186/s13059-014-0523-y.
